# Cell confinement reveals a branched-actin independent circuit for neutrophil polarity

**DOI:** 10.1371/journal.pbio.3000457

**Published:** 2019-10-10

**Authors:** Brian R. Graziano, Jason P. Town, Ewa Sitarska, Tamas L. Nagy, Miha Fošnarič, Samo Penič, Aleš Iglič, Veronika Kralj-Iglič, Nir S. Gov, Alba Diz-Muñoz, Orion D. Weiner

**Affiliations:** 1 Cardiovascular Research Institute and Department of Biochemistry and Biophysics, University of California, San Francisco, California, United States of America; 2 Cell Biology and Biophysics Unit, European Molecular Biology Laboratory, Heidelberg, Germany; 3 Laboratory of Clinical Biophysics, Faculty of Medicine, University of Ljubljana, Slovenia; 4 Department of Theoretical Electrotechnics, Mathematics and Physics, Faculty of Electrical Engineering, University of Ljubljana, Slovenia; 5 Faculty of Health Sciences, University of Ljubljana, Slovenia; 6 Department of Chemical and Biological Physics, Weizmann Institute, Rehovot, Israel; University of Michigan, UNITED STATES

## Abstract

Migratory cells use distinct motility modes to navigate different microenvironments, but it is unclear whether these modes rely on the same core set of polarity components. To investigate this, we disrupted actin-related protein 2/3 (Arp2/3) and the WASP-family verprolin homologous protein (WAVE) complex, which assemble branched actin networks that are essential for neutrophil polarity and motility in standard adherent conditions. Surprisingly, confinement rescues polarity and movement of neutrophils lacking these components, revealing a processive bleb-based protrusion program that is mechanistically distinct from the branched actin-based protrusion program but shares some of the same core components and underlying molecular logic. We further find that the restriction of protrusion growth to one site does not always respond to membrane tension directly, as previously thought, but may rely on closely linked properties such as local membrane curvature. Our work reveals a hidden circuit for neutrophil polarity and indicates that cells have distinct molecular mechanisms for polarization that dominate in different microenvironments.

## Abstract 2

Cells display a high degree of plasticity in migration, but how polarity is organized in different microenvironments has remained unclear. This study uses mechanical perturbations to reveal that migration using actin-rich or bleb-based protrusions are both organized around Rac GTPase.

## Introduction

Directed migration underlies a wide array of biological processes, including embryogenesis, wound healing, and the ability of immune cells to track and destroy pathogens. A key step in migration is polarization, in which cells restrict the activity of their protrusive machinery to a limited portion of their surface. Polarization is thought to arise through the coordinated interactions of short-range positive and long-range negative feedback loops, with actin polymerization playing essential roles in both types of feedback [1–9].

**Fig 1:**
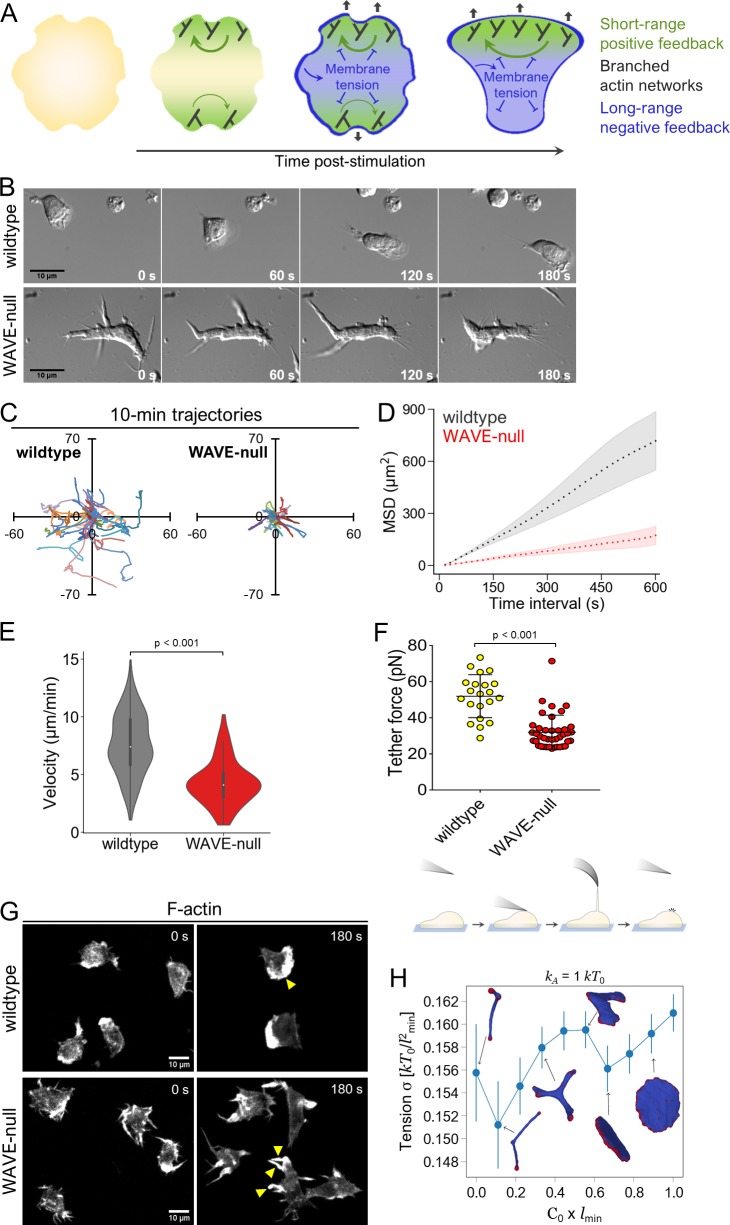
WAVE complex is required for neutrophil polarity and motility. (A) Polarity generation depends on fast-acting, short-range positive feedback and slower-acting, long-range negative feedback. Actin assembly participates in both types of feedback, with its role in negative feedback arising from mechanical force production at the plasma membrane. (B) Migration of dHL-60s on fibronectin-coated glass in uniform chemoattractant (10 nM fMLP) visualized with DIC microscopy. (C–D) dHL-60s were prepared as in B. Cells were tracked using NucBlue nuclear stain and imaged every 15 s by widefield epifluorescence microscopy. (C) Representative tracks from 30 wild-type and 30 WAVE-null cells (randomly selected) over a 10-min observation window. Axes, distance in μm. (D) Mean square displacement versus time interval. Each point represents a time interval that is a multiple of 15 s. Light shaded regions, ±95% CI of the mean. *N* = 273 wild-type and 169 WAVE-null cells pooled from 3 independent experiments. (E) Instantaneous velocity measurements. Each point in the distribution represents the mean instantaneous velocity (calculated over successive 15-s intervals) of a single cell over the 10-min observation window. *N* = 273 wild-type and 169 WAVE-null cells pooled from 3 independent experiments. The same raw data were used to generate this plot and the plot shown in 1D. *p* < 0.001 by unpaired *t* test. (F) Top, membrane tether force was measured using AFM in dHL-60s plated for at least 10 min in 10 nM fMLP. Each point represents a single cell. Data were pooled from 3 independent experiments. Error bars, SD. *p* < 0.001 by unpaired *t* test. Bottom, schematic depicting the experimental procedure. An AFM cantilever was briefly brought into contact with a cell, then withdrawn. In instances when a membrane tether was successfully pulled, the cantilever was maintained at a constant height until the tether broke. The change in cantilever deflection was used to measure the “tether force.” (G) dHL-60s in suspension were fixed and stained for F-actin using rhodamine-phalloidin in the presence or absence of chemoattractant (100 nM fMLP). Yellow arrowheads, actin-rich protrusions. (H) Computational simulations calculating membrane tension σ (see “Methods”) as a function of spontaneous curvature, *c*_*0*_, of actin nucleators. Averaging was performed over 200 statistically independent microstates in equilibrium. Actin nucleators, red. Protein-free lipid bilayer, blue. Error bars, SD. The underlying data for 1C–1F can be found in S1 Data. AFM, atomic force microscopy; dHL-60, differentiated HL-60 cell; DIC, differential interference contrast; fMLP, N-Formylmethionine-leucyl-phenylalanine; WAVE, WASP-family verprolin homologous protein.

When migratory cells first sense a spatially uniform increase in stimulatory cues, they respond by rapidly activating leading-edge polarity factors, including RhoGTPases (e.g., Ras-related C3 botulinum toxin substrate [Rac]), throughout the plasma membrane [7,10]. These GTPases, in turn, activate actin-related protein 2/3 (Arp2/3)-driven assembly of branched actin networks by recruiting nucleation-promoting factors (NPFs) such as WASP-family verprolin homologous protein (WAVE) and Wiskott–Aldrich syndrome protein (WASP) [11–14], resulting in the formation of multiple sheet-like protrusions. Each of these nascent polarity sites is sustained by multiple short-range positive feedback loops (e.g., recruitment of additional RhoGTPase activators [5]), in a manner that often depends on actin assembly [4,15,16]. In parallel with this initial step of activation—but occurring on a slower timescale—cells generate long-range negative feedback to enable a dominant front to emerge [17]. In neutrophils, cells of the innate immune system, this process occurs through the growth of actin filaments, which stretch and increase tension in the plasma membrane [2]. This membrane tension is thought to act as a long-range inhibitor of actin nucleation and polymerization, which enables a “winner-takes-all” competition among the nascent polarity sites to produce a single protrusion (Fig 1A) [2,3,18,19].

While actin polymerization serves as a key ingredient in generating the positive and negative feedback loops that give rise to polarity, we lack an understanding of how specific types of actin networks provide each kind of feedback. Immune cells assemble multiple actin networks at different subcellular locations that carry out distinct functions to support migration: Arp2/3-dependent assembly of branched actin networks at the leading edge contributes to cell guidance/steering and protrusion extension, while actomyosin bundles near the trailing edge provide contractile force to lift the cell rear and squeeze the cell body forward [20,21]. Along with these functional differences, the types of actin networks that immune cells and other migratory cells employ for migration vary with microenvironment [21,22]. The role of actin dynamics in migration is complex and likely depends on the type of actin network, its subcellular location, and the extracellular environment. Existing tools to probe the role of actin networks in both the positive and negative feedback loops needed for polarity are fairly crude and have largely been based on pharmacological perturbations that target all actin polymer [4,6,7,10,18,23,24]. More surgical experiments are needed to clarify how different subcellular actin networks contribute to polarity generation under different environmental conditions.

Here, we address this question by dissecting the role of branched actin assembly in the neutrophil polarity program. We use CRISPR-mediated genome editing in human neutrophils (differentiated HL-60 cells [dHL-60s]) to knock out either the Arp2/3 complex, the nucleator of branched actin assembly [25,26], or its key activator the WAVE complex [27], which promotes branched actin assembly at the leading edge of migratory cells [12,28–31]. We find that during unconfined migration, Arp2/3 and the WAVE complex are each required for the mechanical force generation that supports long-range negative feedback and restriction of protrusion growth to one location. However, each complex is dispensable for polarity and movement of confined cells, such that cell-extrinsic mechanical forces can compensate for the cell-intrinsic forces normally produced by WAVE-dependent actin assembly. This confined movement relies on a completely different mode of leading-edge advance, with processive bleb-based protrusions forming in a back-and-forth motion that extends the leading edge in a serpentine manner. Surprisingly, these serpentine protrusions coincide with Rac-based local positive feedback loops that set a permissive zone for bleb propagation, operate independently of branched actin assembly, and form a mechanistically distinct polarity circuit in this context. Finally, we find that the long-range inhibitor that underlies competition between protrusions does not always respond to membrane tension directly but may rely on closely linked properties such as global cell shape and local membrane curvature. Our data indicate that multiple mechanistically distinct, but conceptually similar, polarity circuits operate in different cell migration contexts.

## Results

### WAVE complex is required for polarization during adhesion-dependent migration

The WAVE complex is required for proper leading-edge formation in immune cells [12,28], and its role in regulating cell shape and motility is broadly conserved across metazoans [32–34]. To generate a human neutrophil cell line lacking the WAVE complex (hereafter referred to as “WAVE-null”), we used CRISPR-Cas9 to knock out *HEM1/NCKAPL1*, which encodes the sole hematopoietic-specific component of the pentameric WAVE complex [12], in dHL-60s. Disruption of *HEM1* is preferable to targeting *WAVE2/WASF2* directly because loss of HEM1 results in the concomitant degradation of other subunits of the WAVE complex, including WAVE2 (Fig A in S1 Fig), and avoids potentially confounding gain-of-function effects of partial subcomplexes [12,28,35].

As expected, WAVE-null (i.e., HEM1-null) cells showed severe polarity and morphological defects when plated in a standard two-dimensional adhesion setting. Treatment of WAVE-null cells with uniform chemoattractant (here and in all other instances, we used the chemoattractant N-Formylmethionine-leucyl-phenylalanine [fMLP]) resulted in the production of dynamic finger-like protrusions radiating from multiple positions on the cell surface rather than a single smooth leading edge typically observed in wild-type cells (Fig 1B and S1 and S2 Videos). WAVE-null cells also showed pronounced motility defects during chemokinesis: when observed over a 10-min period, we found mean square displacement of WAVE-null cells to be significantly lower than that of wild-type cells (Fig 1C and 1D). Similarly, WAVE-null cells migrated more slowly, exhibiting a mean instantaneous velocity of 4.3 ± 0.1 μm/min versus 7.6 ± 0.2 μm/min for wild-type cells (*p* < 0.001, Fig 1E), despite showing similar directional persistence as wild-type cells (Fig B in S1 Fig). The phenotypes we observed are broadly similar to those reported in mouse dendritic cells lacking the WAVE complex [28], in that both cell types failed to form lamellipod-like protrusions at the leading edge. However, WAVE-null mouse dendritic cells showed only mildly impaired motility and appeared to have only a minor polarity defect. These differences may arise from the distinct physiological functions that these two cell types perform [21]. We also note that the polarity defects we observed in WAVE-null neutrophils were more severe than those reported in prior work using RNA interference (RNAi) to knock down the HEM1 subunit of the WAVE complex [12]. As our CRISPR-based approach eliminates all protein, the milder defects observed upon HEM1 knockdown may arise from residual WAVE complex following RNAi.

The multipolar phenotype we observed in WAVE-null neutrophils could arise from a defect in membrane tension generation, which may be needed for producing the negative feedback that fronts use to compete with one another [2,36] (Fig 1A). To test this idea, we used atomic force microscopy (AFM) to measure membrane tether force in cells stimulated with chemoattractant. WAVE-null cells showed a 2-fold decrease in tether force compared with wild-type cells (Fig 1F), corresponding to a 4-fold decrease in membrane tension when using a common model relating tether force to membrane tension ([2,18]; see [37] for calculation details). These decreases were observed despite WAVE-null cells forming multiple actin-rich protrusions in response to stimulation with chemoattractant (Fig 1G). Our results show that WAVE-dependent actin assembly is required for proper levels of membrane tension following stimulation with chemoattractant.

We further performed computational simulations using a simplified system containing membrane curvature-sensing actin nucleators in a vesicle to assess whether changes in the spatial organization of actin nucleation can correlate with changes in membrane tension [38] (Fig A in S2 Fig; see “Methods” for details). Note that in cells, the curvature sensitivity and the actin nucleation activity may be performed by distinct proteins that form a bound complex at the membrane [39]. Proteins in our model represent such complexes as a single entity, for simplicity. In this model, each membrane protein has a specified spontaneous curvature and is free to diffuse and bind to other proteins, forming aggregates of different sizes and shapes. In addition, since each such protein represents a site of actin nucleation, it exerts an outwards protrusive force. By modulating the curvature preference of a fixed concentration of actin nucleators, we found that the membrane spontaneously deforms into shapes ranging from those containing multiple finger-like protrusions (similar to WAVE-null cells) to a single sheet-like protrusion (similar to wild-type cells). Transition in membrane shape from finger-like to sheet-like coincided with small increases in membrane tension (Fig 1H and Fig B in S2 Fig). These simulations show that, even if the concentration of actin nucleators remains constant, changes in their organization can strongly affect the morphology of the resulting protrusions they form and, consequently, the amount of membrane tension they can generate. In combination with our experimental data, they suggest that spatial organization of the WAVE complex at the plasma membrane may contribute to the WAVE complex’s ability to generate lamellipod-like protrusions.

### Key leading-edge polarity factors require Arp2/3 and WAVE complex for proper regulation

**Fig 2:**
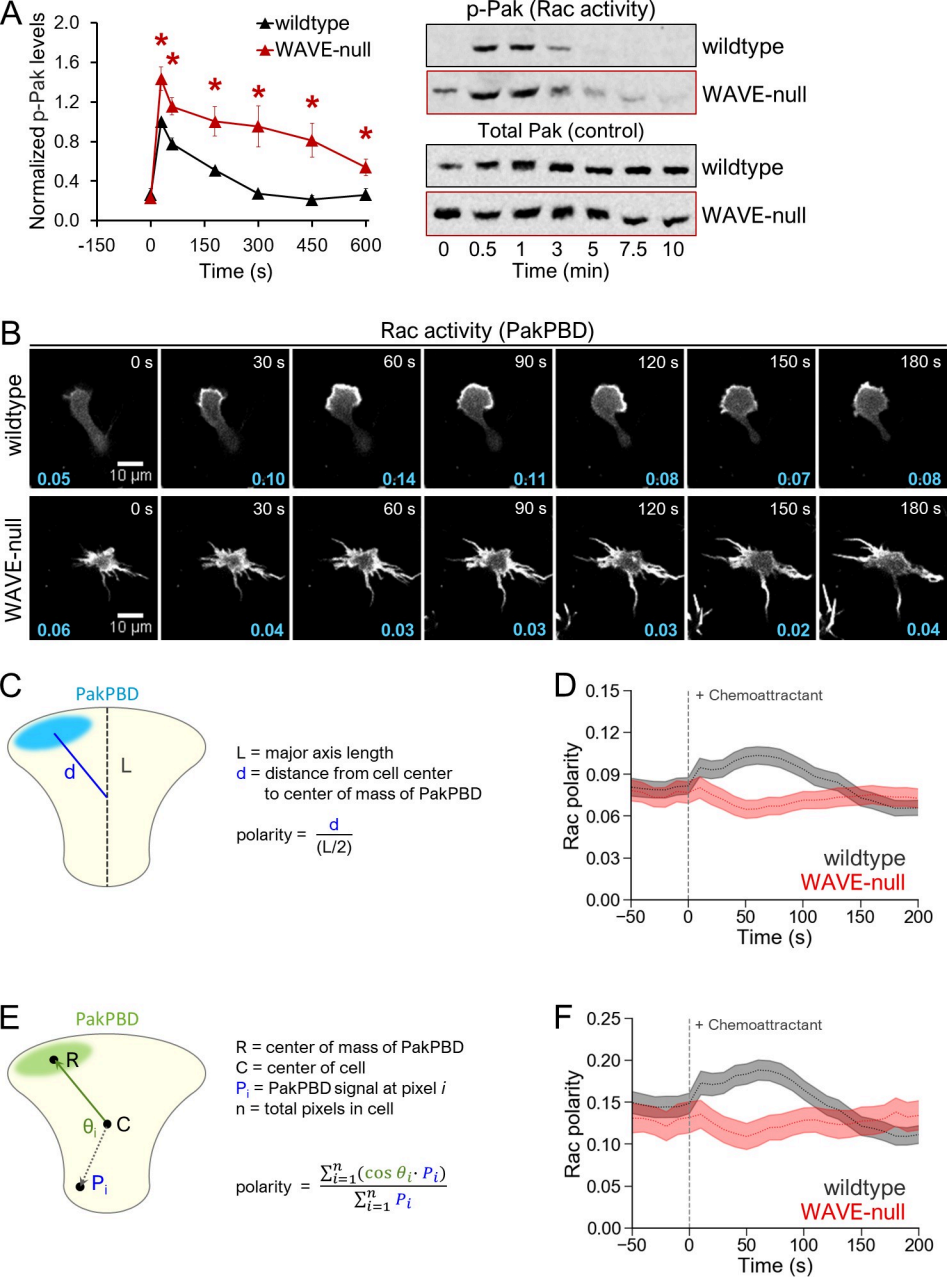
Loss of WAVE complex results in aberrant levels and polarization of Rac activity. (A) Rac activity was quantified for chemoattractant-stimulated cells using antibodies targeting phospho-Pak, a downstream readout of Rac activation. Antibodies targeting total Pak were used as loading controls (see the “Immunoblot assays” section of “Methods” for details). Left, each point represents an average of 4 independent experiments, with data for each experiment normalized to wild-type cells at “30 s,” reported as “1.0.” Error bars, SEM. *p < 0.05 by unpaired *t* test. Right, representative immunoblot. (B) dHL-60s expressing the Rac biosensor PakPBD-mCitrine were plated on fibronectin-coated glass, stimulated with 10 nM fMLP, and imaged by confocal microscopy every 10 s. Values in cyan indicate the degree of PakPBD polarization, as described in D. (C, E) Schematic depicting calculation of Rac activity polarity using (C) normalized distance or (E) angular distribution. See “Methods” for details. (D, F) dHL-60s were prepared as in 2B, and polarity of Rac activity was measured for single cells at each 10-s interval using (D) normalized distance (*N* = 295 wild-type and 218 WAVE-null cells pooled from 3 independent experiments) or (F) angular distribution (*N* = 231 wild-type and 141 WAVE-null cells pooled from 3 independent experiments). Dashed lines, mean polarity of Rac activity. Light shaded regions, ±95% CI of the mean. The underlying data for 2A, 2D, and 2F can be found in S1 Data. dHL-60, differentiated HL-60 cell; fMLP, N-Formylmethionine-leucyl-phenylalanine; PakPBD, p21-activated kinase binding domain; Rac, Ras-related C3 botulinum toxin substrate; WAVE, WASP-family verprolin homologous protein.

Membrane tension is thought to play a major role in negatively regulating the polarity factors that organize protrusion growth, including the RhoGTPase Rac [2,40]. The morphological polarity defects observed in WAVE-null cells are expected based on the decreased membrane tension in these cells, which should fail to engage the global negative feedback circuit that constrains the amount and spatial distribution of Rac activity. We tested this hypothesis by monitoring the levels of Rac activation using the phosphorylation state of p21-activated kinase (Pak) kinase, a Rac effector [41]. Following stimulation, WAVE-null cells produced significantly higher levels of phospho-Pak than wild-type cells (Fig 2A), consistent with elevated levels of Rac activity. To control for off-target effects of CRISPR-mediated genome editing, we generated an independent clonal cell line, “WAVE-null, clone 2,” (Fig E in S1 Fig) which similarly showed elevated phospho-Pak levels compared with wild-type cells (Fig F in S1 Fig). These results are also in agreement with recent work revealing a role for actin polymerization in negatively regulating Rac activity in neutrophils [42].

We next examined whether elevated levels of Rac activity coincided with failure to restrict Rac activity to a single site (i.e., loss of polarity, by which we mean loss of unipolarity in this context). Using the p21-activated kinase binding domain (PakPBD) biosensor [7,43], we monitored the spatial distribution of Rac activity in live cells. Stimulation of wild-type cells with uniform chemoattractant produced an initial burst of Rac activity throughout the plasma membrane, followed by its restriction to a single protrusion (Fig 2B, top row; S3 Video), in agreement with prior work [7,10]. WAVE-null cells, in contrast, maintained multiple protrusions enriched for Rac activity for the entire time course (Fig 2B, bottom row; S3 Video) and failed to achieve polarity. We quantified Rac activity polarity (i.e., spatial restriction to a single focus) in these cells by measuring the distance between the geometric cell center and the center of weighted Rac activity signal, followed by normalization using the cell length (Fig 2C). This yields a “Rac polarity” score from 0 to 1, with a value of 0 indicating multiple foci of Rac activity distributed uniformly around the cell and a value near 1 indicating a single focus of Rac activity on the portion of the membrane furthest from the cell center (see “Methods” for details). Populations of wild-type neutrophils polarized Rac activity in response to uniform chemoattractant, with cells reaching peak polarity around 60 s (Fig 2D). In contrast, WAVE-null cells showed no Rac polarity increase following stimulation with chemoattractant (Fig 2D), consistent with impaired spatial regulation of Rac activity.

To assess whether this normalized distance method for determining polarity is robust, we compared it with an alternative orthogonal method [44] relying on angular distribution of Rac activity about the geometric cell center (Fig 2E, see “Methods” for details). This method similarly yields a Rac polarity score from 0 to 1, with 0 indicating multiple foci of Rac activity (i.e., PakPBD) with a uniform angular distribution around the geometric cell center and 1 indicating that all of the Rac activity lies on a single vector originating from the cell center. When re-quantifying our original data using this angular distribution method (Fig 2F), we found that the results closely paralleled our normalized distance method (Fig 2D) and confirmed that differences in polarity between wild-type and WAVE-null cells can be observed using multiple metrics. For the remainder of our experiments, we used our normalized distance method for quantifying polarity.

**Fig 3:**
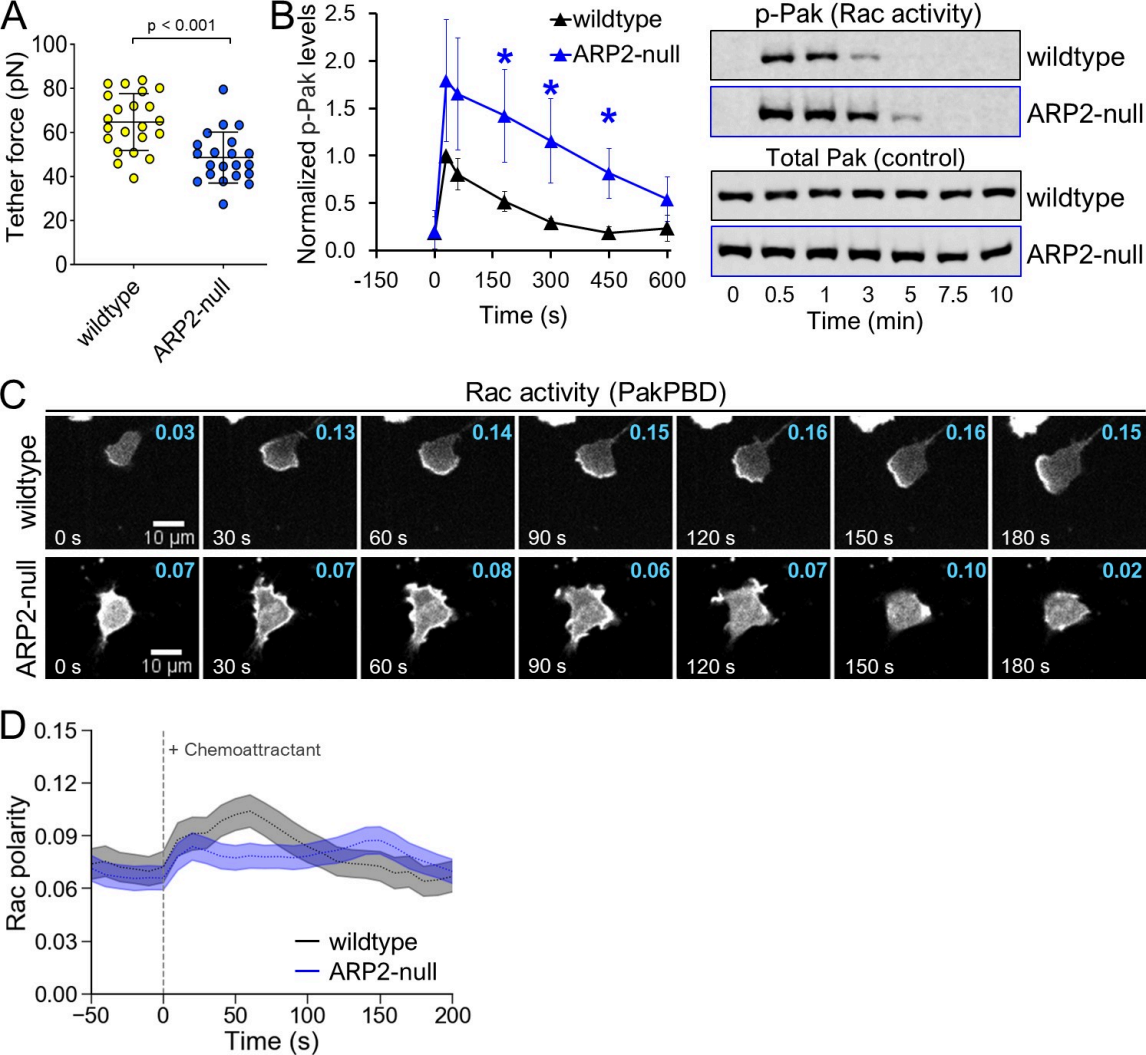
Loss of Arp2/3 complex phenocopies polarity defects observed in WAVE-null cells. (A) Membrane tether force of dHL-60s was measured using AFM as described in 1F. Each point represents a single cell. Data were pooled from 3 independent experiments. Error bars, SD. *p* < 0.001 by unpaired *t* test. (B) dHL-60s were stimulated with 10 nM fMLP, and samples were collected and processed for immunoblot. Left, Rac activity was indirectly quantified as in 2A. Each point represents an average of 4 independent experiments, with data for each experiment normalized to wild-type cells at 30 s. Error bars, SEM. *p < 0.05 by unpaired *t* test. Right, representative immunoblot. (C) dHL-60s expressing PakPBD-mCitrine were imaged as in 2B. Values in cyan indicate the degree of PakPBD polarization, as described in 2C. (D) Quantification of Rac polarity as described in 2C for cells prepared as in 3C. Dashed lines, mean Rac polarity. Light shaded regions, ±95% CI of the mean. *N* = 151 wild-type and 228 ARP2-null cells pooled from 3 independent experiments. The underlying data for 3A-B and 3D can be found in S1 Data. AFM, atomic force microscopy; Arp2/3, actin-related protein 2/3; dHL-60, differentiated HL-60 cell; fMLP, N-Formylmethionine-leucyl-phenylalanine; PakPBD, p21-activated kinase binding domain; Rac, Ras-related C3 botulinum toxin substrate; WAVE, WASP-family verprolin homologous protein.

A major cellular function of the WAVE complex is stimulation of Arp2/3-dependent branched actin assembly, but the WAVE complex’s ability to regulate Rac could also stem from its additional roles. In other systems, the WAVE complex interacts with regulators of Rac (e.g., Rac GTPase-activating proteins [GAPs] [45]) or additional actin assembly factors (e.g., formins [46]). Furthermore, the WAVE complex may contribute to the growth of actin networks nucleated by WAVE-independent processes [47]. To test whether such Arp2/3-independent functions of the WAVE complex may contribute to its role in Rac regulation, we performed additional experiments in cells lacking functional Arp2/3 complex (Fig C in S1 Fig). These ARP2-null cells phenocopied the key defects that we observed in WAVE-null neutrophils: ARP2-null cells showed impaired membrane tension generation (Fig 3A), elevated levels of Rac activity (Fig 3B), and diminished polarization of Rac activity in response to stimulation with uniform chemoattractant (Fig 3C and 3D; S3 Video). These effects were similar to wild-type cells treated with CK-666 to acutely block Arp2/3 activity: we previously found cells treated with CK-666 to have impaired membrane tension generation [18], and here we found them to also have elevated levels of Rac activity (Fig D in S1 Fig). However, unlike WAVE-null cells, ARP2-null cells stimulated with chemoattractant did not produce elongated finger-like protrusions (S4 Video), suggesting that such protrusions in WAVE-null cells may depend on Arp2/3 activity. These data are consistent with the WAVE complex primarily regulating polarity of Rac activity through its stimulation of branched actin assembly rather than through its other cellular functions.

### Hypotonic treatment restores polarity in the absence of WAVE complex

**Fig 4:**
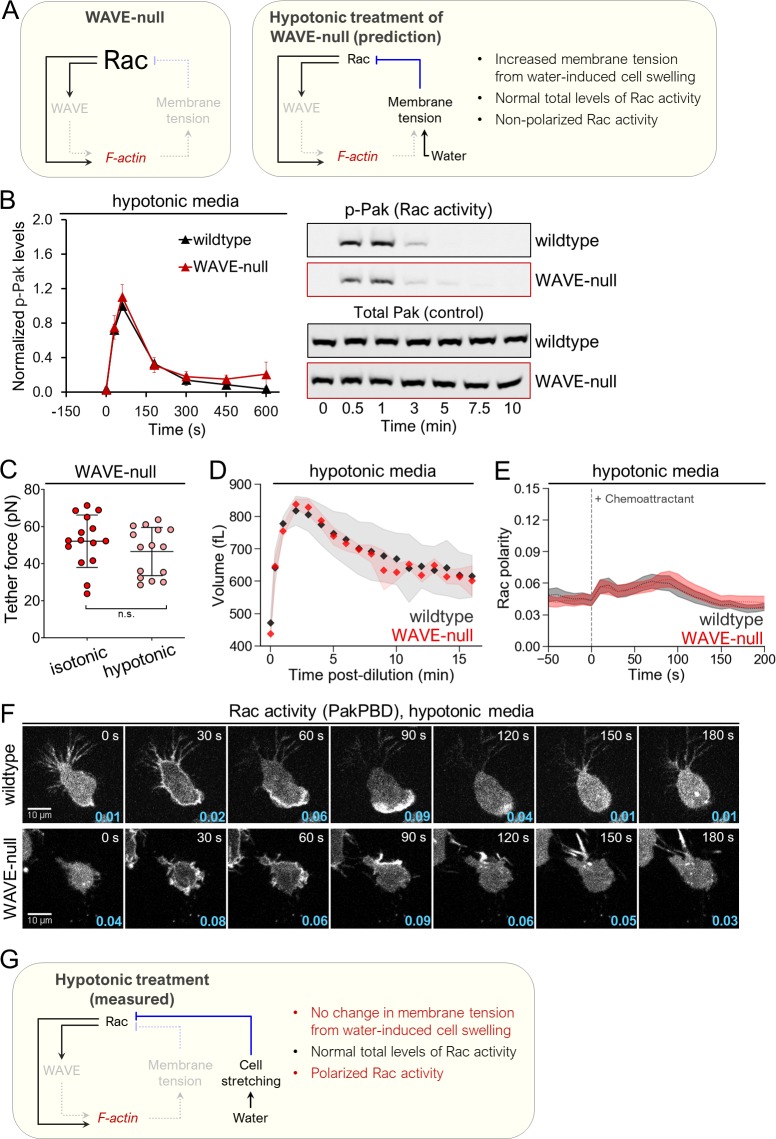
Hypotonic treatment to extend the plasma membrane restores spatiotemporal Rac activity in cells lacking WAVE complex. (A) Left, schematic depicting key components in the neutrophil polarity circuit and their measured alterations in WAVE-null cells. Right, expected regulatory changes among key polarity components in WAVE-null cells following hypotonic-induced plasma membrane stretching, where we hypothesized a rescue of Rac activity levels. (B) dHL-60s were suspended in 0.5× isotonic media (see “Methods” for details), stimulated with 10 nM fMLP, and samples were collected and processed for immunoblot. Left, Rac activity was quantified via phospho-Pak as in 3B. Each point represents an average of 4 independent experiments, with data for each experiment normalized to wild-type cells at 30 s. Error bars, SEM. Right, representative immunoblot. (C) Membrane tether force of dHL-60s was measured using AFM as described in 1F. The same cells were measured before and after hypotonic shock. Note that the cells in the isotonic condition are a subset of the data depicted in 1F that were further subjected to hypotonic treatment. The data are reproduced here to aid comparison. Each point represents a single cell. Data were pooled from 4 independent experiments. Error bars, SD. Membrane tension does not significantly change following hypotonic shock (*p* > 0.05 by paired *t* test). (D) Volumes of dHL-60s in suspension following hypotonic treatment (0.5× isotonic media) were measured using a Coulter Counter. Each point represents the mode volume for a distribution of >7,000 cells (see “Methods” for details) averaged from 4 independent experiments. Light shaded regions, ±SD. Volumes of wild-type and WAVE-null cells in isotonic media (i.e., at 0 min) do not differ significantly (*p* = 0.09 by unpaired *t* test). Volume significantly increases following hypotonic shock (*p* < 0.01 by paired *t* test when comparing volumes of wild-type or WAVE-null cells at 0 min versus 16 min). (E) Quantification of Rac polarity as described in 2C for cells prepared as in 4F. Dashed lines, mean polarity of Rac activity. Light shaded regions, ±95% CI of the mean. *N* = 204 wild-type and 177 WAVE-null cells pooled from 3 independent experiments. (F) dHL-60s in hypotonic media (0.5× isotonic) expressing the Rac activity reporter PakPBD-mCitrine were imaged as in 2B. Values in cyan indicate the degree of Rac polarization, as described in 2C. (G) Schematic depicting measured regulatory changes among key polarity components in WAVE-null cells following hypotonic treatment. Compare with expected changes in 4A. The underlying data for 4B-E can be found in S1 Data. dHL-60, differentiated HL-60 cell; PakPBD, p21-activated kinase binding domain; Rac, Ras-related C3 botulinum toxin substrate; WAVE, WASP-family verprolin homologous protein.

If the WAVE complex primarily regulates long-range inhibition of leading-edge signals by imparting mechanical force on the plasma membrane, we should be able to restore long-range inhibition by providing compensatory mechanical forces. One approach for mimicking WAVE’s extension of the plasma membrane is to place neutrophils in hypotonic media, where osmotic-based cell swelling may provide such a force (Fig 4A) [2,48]. Because wild-type neutrophils can still polarize and migrate in 0.5× isotonic media (i.e., a 1:1 dilution of isotonic media with deionized water) [18], we examined how stimulating WAVE-null cells under similar conditions affected the amount of Rac activity. In hypotonic media (0.5× isotonic), both wild-type and WAVE-null cells showed nearly identical levels of Rac activity throughout the entire time course (as assayed via Pak phosphorylation, Fig 4B), indicating a rescue of the Rac activity defect observed in WAVE-null cells in isotonic media (Fig 2A). These data suggest that hypotonic treatment can supply the mechanical force that is normally provided by WAVE-dependent actin assembly to generate the negative feedback needed for limiting total cellular Rac activity.

We next examined what physical changes the WAVE complex might impart on the plasma membrane to regulate Rac activity. Prior work concluded that membrane tension increases play a primary role in this regulatory behavior: Experimental perturbations that increase membrane tension lead to inhibition of both Rac activity and the WAVE complex; conversely, decreases in membrane tension lead to their activation throughout the cell [2]. However, tension increases frequently coincide with other physical changes (e.g., unfolding of membrane reservoirs [19,49]), and it is not clear whether membrane tension itself or a close correlate forms the basis of the long-range inhibition. Determining whether membrane tension or other physical parameters execute the long-range inhibition is critical to identifying the relevant mechanosensor that orchestrates this process [50]. Using AFM to measure membrane tether force, we found that, similar to wild-type cells [18], mild hypotonic treatment of WAVE-null cells did not increase membrane tension (Fig 4C), so the rescue of Rac activity by hypotonic treatment is not a consequence of increased membrane tension. However, hypotonic shock did cause other alterations to the organization of the membrane of WAVE-null cells that normally go along with tension changes. In particular, volume increased by nearly 2-fold (*p* < 10^−8^ for cells at 0 min versus 2 min by paired *t* test) (Fig 4D), indicating a significant increase in the apparent surface area of the plasma membrane [49]. These observations are consistent with leukocytes maintaining membrane reservoirs that can be released in response to osmolarity decreases [51,52]. Furthermore, they carry the surprising implication that polarity-regulating mechanosensors are likely to not exclusively respond to membrane tension alone but may depend on other physical changes in the plasma membrane.

As hypotonic treatment resulted in both wild-type and WAVE-null cells producing similar amounts of total Rac activity, we assessed whether this perturbation might also restore polarization of Rac activity in WAVE-null cells using quantification of PakPBD localization as previously described (Fig 2C). We found that WAVE-null cells in 0.5× isotonic media could mildly polarize Rac activity in response to chemoattractant (Fig 4E and 4F; S5 Video), whereas they had failed to do so in isotonic media (Fig 2B and 2D). However, hypotonic treatment did not affect protrusion morphology, which remained finger-like (Fig 4F, bottom row). Our results show that other mechanisms may function independent of the WAVE complex to polarize Rac activity, so long as the requirement for long-range inhibition (i.e., mechanical force) is satisfied—in this case by hypotonic-induced cell swelling (Fig 4G).

### Arp2/3 and WAVE complex are dispensable for polarization in confined environments

**Fig 5:**
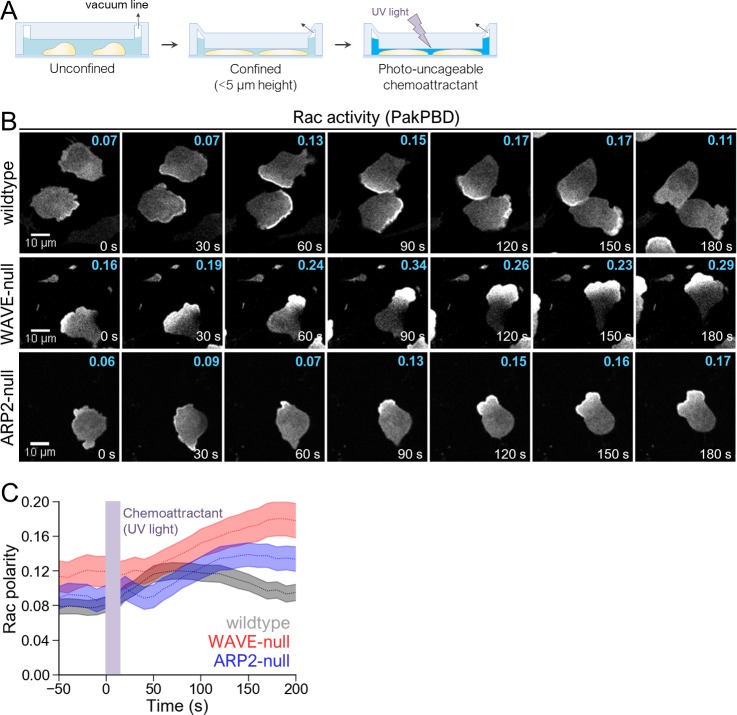
Cell compression restores polarization in cells lacking WAVE or Arp2/3 complex. (A) Schematic for cell confinement experiments. The height of the chamber was set using a vacuum regulator. (B) dHL-60s expressing the Rac biosensor PakPBD were plated on fibronectin-coated glass in media containing 10 μM caged fMLP and imaged every 10 s by confocal microscopy. Chamber height was set as shown in 5A. Values in cyan indicate the degree of Rac activity polarization, as described in 2C, for the topmost cell fully inside each panel. (C) Quantification of Rac polarity as described in 2C for cells prepared as in 5B. Violet bar indicates when UV was used to photo-uncage caged fMLP (0–20 s). Dashed lines, mean polarity of Rac activity. Shaded regions, ±95% CI of the mean Rac polarity. *N* = 171 wild-type cells pooled from 4 independent experiments, 101 WAVE-null cells pooled from 4 independent experiments, and 108 ARP2-null cells pooled from 3 independent experiments. The underlying data for 5C can be found in S1 Data. Arp2/3, actin-related protein 2/3; dHL-60, differentiated HL-60 cell; fMLP, N-Formylmethionine-leucyl-phenylalanine; PakPBD, p21-activated kinase binding domain; Rac, Ras-related C3 botulinum toxin substrate; WAVE, WASP-family verprolin homologous protein.

Because hypotonic treatment has the potential to introduce pleiotropic effects beyond cell shape changes, we employed an orthogonal approach to stretch cells using mechanical force. Using polydimethylsiloxane (PDMS)-based devices, we created confined environments in which chamber height could be adjusted by applying vacuum [53]. We initially confined neutrophils by adjusting the height of the chamber to <5 μm. Acute stimulation of cells in this device was achieved using UV light to release chemically caged chemoattractant [54,55] (Fig 5A), and polarization of Rac activity was monitored using PakPBD. As expected, wild-type cells under confinement produced chemoattractant-based increases in polarized Rac activity (Fig 5B and 5C; S6 Video). These responses broadly resembled those we observed in wild-type cells during unconfined migration (Fig 2B and 2D).

Whereas unconfined WAVE-null cells failed to polarize Rac activity when stimulated, confinement gave a profound rescue of polarity. Cells switched from producing finger-like protrusions to generating smooth leading edges that much more closely resembled the overall shape of wild-type cells (Fig 5B, middle row; S6 Video). These alterations in polarity and morphology were rapid and reversible. Repeatedly raising and lowering the height of the chamber (to alter the degree of confinement) caused WAVE-null neutrophils to switch from generating clustered finger-like protrusions to making smooth protrusions (S7 Video). Stimulation of these confined WAVE-null neutrophils resulted in pronounced polarization of Rac activity that was even more profound than in wild-type cells under this degree of confinement (Fig 5C). Polarization of confined cells depended on acute increases in chemoattract, as wild-type cells not treated with UV light (i.e., chemoattractant remained photocaged throughout imaging) did not polarize (S3 Fig). Experiments performed in ARP2-null cells produced similar results (Fig 5B and 5C), further underscoring that all branched actin assembly is dispensable for supporting Rac polarity, leading-edge morphology, and persistent motility in confined environments.

We performed additional experiments under conditions in which cells were only weakly confined (chamber height of 5–9 μm). While wild-type, WAVE-null, and ARP2-null cells all exhibited polarized Rac activity when stimulated with chemoattractant, we found that WAVE-null cells did not polarize Rac activity as strongly as wild-type or ARP2-null cells (Fig A-C in S4 Fig, S6 Video). Furthermore, WAVE-null cells at this confinement regime generated finger-like rather than smooth protrusions (Fig B in S4 Fig, middle row). Our observations in weakly confined WAVE-null cells are similar to those in hypotonically treated WAVE-null cells (Fig 4F, bottom row), where polarized Rac activity was rescued despite cells still forming protrusions of aberrant morphology. These results show that polarization of key leading-edge factors can be decoupled from protrusion morphology and may serve as a useful platform in future studies focusing on the mechanisms underlying each.

We then performed computational simulations to assess what physical processes might underlie the ability of WAVE-null cells to form smooth protrusions under compression. Using the system described in Fig 1H ([38] see also “Methods”), we observed how a fixed concentration of actin nucleators having zero (or small) spontaneous curvature (which produced vesicles with finger-like protrusions in an unconfined setting) affected vesicle morphology in response to varying degrees of compression. Similar to the observations of confined cells, we found that increasing levels of confinement for our computational simulations (lower values of *d/l*_*min*_) resulted in vesicles converting from a morphology of primarily finger-like protrusions to one containing a single sheet-like protrusion (Fig C-E in S2 Fig, S8 and S13 Videos). However, our computational simulations do not explicitly include the effects of myosin contractility, which was found to be required for these WAVE-null cells to produce smooth protrusions while under confinement (see next section, “Polarization under confinement relies on processive bleb-based protrusions in the absence of WAVE”). Furthermore, the smooth protrusions of these cells have bleb-like characteristics, and blebbing is driven by myosin contractility. Nevertheless, it is of interest to compare to the transition we find within our simple model, as it is insightful and relevant to know how the interplay between membrane bending energy and protrusive forces affects membrane morphology, which may appear in other cellular contexts in which protrusion dominates (see S1 Text for more detailed discussion).

### Polarization under confinement relies on processive bleb-based protrusions in the absence of WAVE

**Fig 6:**
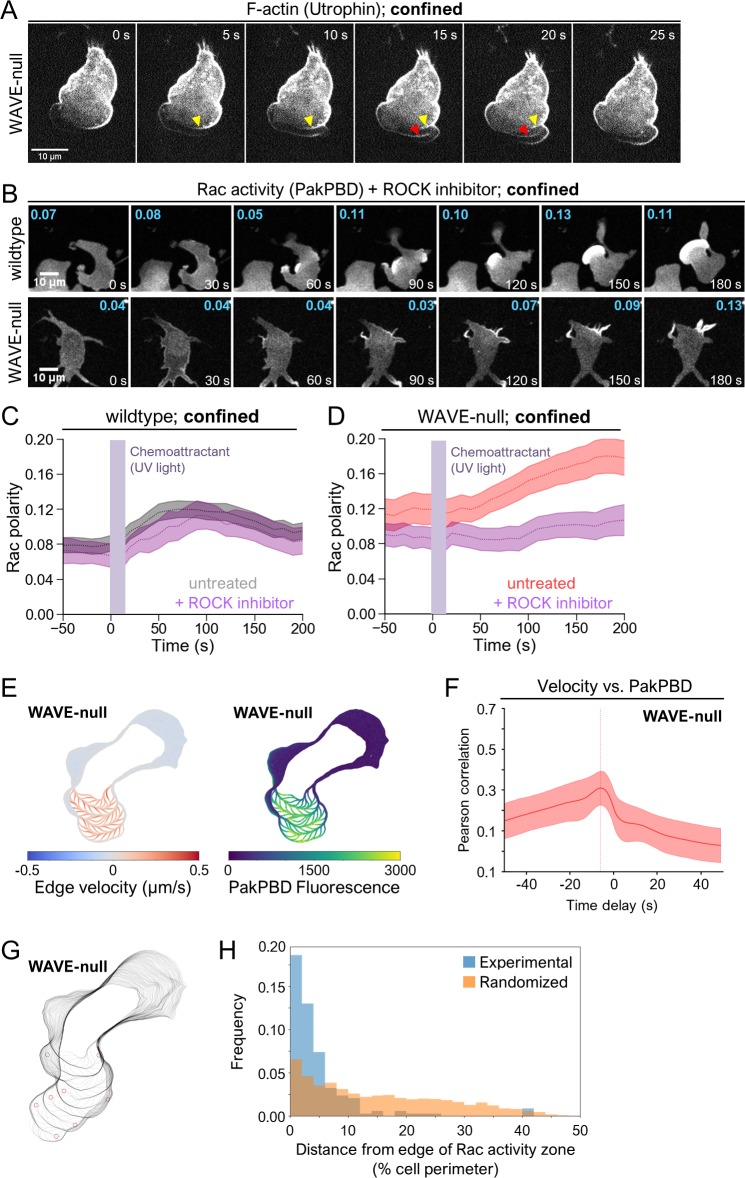
WAVE-null cells under confinement migrate using bleb-like protrusions. (A) dHL-60s expressing the F-actin marker Utr261-mCherry were plated on fibronectin-coated glass in media containing 10 μM caged fMLP and imaged every 1 s by confocal microscopy. Prior to imaging, cells were confined as in 5A and fMLP was photo-uncaged. Yellow and red arrows indicate where cortical actin networks are left behind following formation of a bleb. (B) dHL-60s expressing the Rac biosensor PakPBD were plated on fibronectin-coated glass in media containing 10 μM caged fMLP and 20 μM Y27632 and imaged as in 5B. Prior to imaging, cells were confined as in 5A. Values in cyan indicate the degree of Rac polarization, as described in 2C, for the topmost cell fully inside each panel. (C–D) Quantification of PakPBD polarity as described in 2C for cells prepared as in 6A. Violet bars indicate when UV was used to photo-uncage caged fMLP (0–20 s). Dashed lines, mean polarity of Rac activity. Light shaded regions, ±95% CI of the mean Rac polarity. (C) *N* = 51 Y27632-treated wild-type cells pooled from 3 independent experiments. Data for “untreated” cells are reproduced from wild-type cells in 5C to aid in comparison. (D) *N* = 81 Y27632-treated WAVE-null cells under confinement pooled from 3 independent experiments. Data for “untreated” cells are reproduced from WAVE-null cells in 5C to aid in comparison. (E) Overlays of extracted cell boundaries over time displaying associated edge velocity (left) and PakPBD edge fluorescence (right). (F) Analysis of Pearson correlation between edge velocity and PakPBD fluorescence as a function of temporal offset in fluorescence. The peak Pearson correlation occurs when fluorescence values of Rac activation are shifted back in time by 6 s relative to membrane extension, indicating that membrane extension precedes changes in local Rac activation by 6 s. Red line and shaded area, mean ±95% CI. *N* = 35 WAVE-null cells pooled from 3 independent experiments. (G) Overlays of extracted cell boundaries as in 6E with red circles marking locations of computationally identified reversals in bleb propagation direction. (H) Analysis of spatial coincidence of bleb reversals and boundaries of Rac activity zone. To generate the randomized distribution (orange), the Rac activity zone was rotated randomly, and the minimal distances were recalculated. This randomization process was repeated 20 times and the results pooled. *N* = 169 reversals. The underlying data for 6C-D, 6F, and 6H can be found in S1 Data. dHL-60, differentiated HL-60 cell; fMLP, N-Formylmethionine-leucyl-phenylalanine; PakPBD, p21-activated kinase binding domain; Rac, Ras-related C3 botulinum toxin substrate; Utr261, utrophin 261; WAVE, WASP-family verprolin homologous protein.

In addition to using actin-rich pseudopods for motility, migratory cells build bleb-based protrusions in certain microenvironments [21,22]. These types of protrusions depend on high actomyosin contractility and occur when local weakening of membrane-to-cortex attachments (MCAs) leads to the separation of the plasma membrane from the underlying actin cortex. Hydrostatic pressure then drives the expansion of this detached segment of membrane to form a protrusion initially lacking filamentous actin [56]. Because WAVE-null cells are impaired in branched actin assembly at the leading edge, we asked whether they may instead rely on blebbing to generate polarity under confinement. For this purpose, we investigated leading-edge dynamics in confined WAVE-null cells expressing an actin-binding fragment of utrophin 261 (Utr261), which associates with cortical actin networks in neutrophils [11]. Formation of a bleb generally results in a protrusion initially lacking any actin filaments [57], and, consistent with this behavior, confined WAVE-null cells exhibited leading edges with portions of the cytosol extending beyond the boundary of the cortical actin network (Fig 6A). Intriguingly, the bleb-based protrusions we observed in WAVE-null cells occurred via processive extensions of the plasma membrane that travelled around the cell periphery. At regular intervals, the extension would reverse directions and form a highly stereotyped serpentine pattern, confining processive extensions to a small section of the plasma membrane to form a leading edge (S9 Video). This mode of protrusion extension resembles long-known “circus movements,” in which a bleb forms and progressively extends around the cell in a clockwise or counterclockwise direction [58]. However, circus movements only occasionally undergo direction reversals [59], whereas serpentine protrusions in confined WAVE-null neutrophils do so frequently, on the order of seconds.

As blebbing is powered by actomyosin contractility, we next asked whether the serpentine protrusions of confined WAVE-null cells showed a similar requirement for myosin activity. We treated neutrophils with the Rho-associated protein kinase (ROCK) inhibitor Y27632 to block myosin contractility [60]. In other contexts, ROCK is required for blebbing but is dispensable for actin protrusion-based chemotaxis [61]. Confined wild-type neutrophils treated with Y27632 showed little change in polarized Rac activity, consistent with protrusion growth and cell movement being independent of actomyosin-based contractility in these cells (Fig 6B and 6C). In contrast, WAVE-null cells treated with Y27632 under confinement showed reduced polarization of Rac activity upon stimulation and formed only finger-like protrusions (Fig 6B and 6D). However, Y27632-treated WAVE-null cells under confinement still exhibited greater polarity than unconfined WAVE-null cells (Fig 2D), suggesting that other processes independent of myosin contractility contribute to polarization of Rac activity. Together, our data show that protrusion generation in confined WAVE-null cells arises from actomyosin-dependent blebbing.

### A potential Rac-mediated positive feedback circuit for bleb-based polarization

Established models describing polarization specify the initial production of short-range positive feedback that amplifies small fluctuations in polarity factor activity and long-range negative feedback that acts to limit the spread of the positive feedback and suppress secondary fronts. The combined actions of these two types of feedback lead to the consolidation of polarity to a single site [1]. Along with the role of actin polymerization in generating long-range inhibition [2,62], there is also a well-established requirement for actin assembly in mediating short-range positive feedback during polarization of migratory cells (Fig 1A, green arrows) [4–7,10,23,24]. The highly polarized Rac activity that we observed in confined WAVE-null cells suggested the presence of short-range positive feedback loops in maintaining localized protrusion growth, despite their impaired branched actin assembly. This observation suggests that the mechanical forces resulting from cell confinement may provide negative feedback that leads to spatial restriction of Rac activity to a small portion of the plasma membrane. To explore this possibility further, we imaged cells expressing PakPBD with high temporal frequency to assess whether Rac activity correlated with growth of blebs. Confined WAVE-null cells showed high levels of Rac activity coinciding with the location of serpentine protrusions (i.e., the leading edge) (S10 Video). To investigate the relation of Rac activity and blebbing more quantitatively, we performed a cross-correlation analysis in which we divided the plasma membrane into 1,000 discrete segments and quantified both edge velocity and PakPBD fluorescence for each membrane segment over time (Fig 6E and S11 Video; see “Methods” for details). Edge velocity and PakPBD showed an average instantaneous Pearson correlation coefficient of 0.17 (Fig 6F). We next shifted the PakPBD intensity measurements for a given timepoint relative to the edge velocity measurements to assess whether local increases in Rac activity came before or after local increases in edge velocity. The correlation between PakPBD fluorescence and edge velocity was highest (Pearson correlation coefficient of 0.31) when PakPBD signal was shifted backwards in time by 6 s relative to edge velocity (Fig 6F). We obtained similar results when performing this analysis with wild-type cells (Fig A in S5 Fig, maximum Pearson correlation coefficient of 0.33 at a time delay of −9 s). Confined wild-type cells migrated primarily using smooth lamellipod-like protrusions, but occasionally formed transient bleb-based protrusions (S12 Video). This analysis indicates that the localized extension of bleb-based protrusions slightly precedes Rac activation and further suggested that Rac activity may restrict the region of the plasma membrane that is permissive for protrusion extension. To test this idea, we computationally identified locations in which processive blebbing events underwent reversals (e.g., Fig 6G and Fig B in S5 Fig) and determined the distance (as a percentage of total cell perimeter) between reversal location and the edge of the Rac activity zone (see “Methods” for details). The median distance at which reversals occurred, within 3% ± 7% of the Rac activity zone edge, differed substantially from a distribution in which reversals occur at random sites along the cell perimeter, within 13% ± 12% of the Rac activity zone edge (Fig 6H). These data are consistent with Rac activity providing a permissive zone for bleb propagation. In conjunction with our observation that blebbing also precedes Rac activation, they also indicate a bleb-based positive feedback circuit for Rac regulation. These data indicate that neutrophils employ additional feedback mechanisms beyond branched actin assembly to enrich Rac activity at the leading edge. In future work, the ability of confined WAVE-null cells to migrate exclusively using bleb-based protrusions may serve as a useful platform for dissecting the short-range positive feedback loops underlying bleb-based migration.

## Discussion

Polarization of migratory cells requires coordinated positive and negative feedback loops to restrict protrusion growth to a single site. Actin polymerization is thought to underlie both types of feedback [4,6,7,10,18,23,24], but the roles of different types of actin networks in providing each kind of feedback have not been carefully addressed. Here, we show that branched actin assembly is dispensable for producing short-range positive feedback of Rac activation (Figs 4E, 4F, 5B and 5C), whereas the importance of actin in providing long-range negative feedback varies with microenvironment (compare Figs 2A and 3B with Fig 4B). These observations require a revision of the dominant view that branched actin assembly plays essential roles in both types of feedback. Our work further demonstrates how combined genetic and mechanical perturbations can be used to decouple different migration modes and isolate the components regulating each.

For the long-range inhibition that enables fronts to compete with one another, our data show that this negative feedback can be supported by mechanical deformation of the plasma membrane (Fig 4). When neutrophils migrate in unconfined environments, WAVE-dependent branched actin assembly is required for generating this force (Figs 1 and 2). However, upon confinement, the resulting membrane extensions arising from this perturbation can compensate for the polymerization force normally provided by branched actin assembly (Fig 5). This confinement puts WAVE-null cells in a regime in which producing either finger-like actin-rich protrusions or bleb-based protrusions may now provide enough protrusive force to satisfy the long-range negative feedback requirement for polarity. However, the degree to which bleb-based protrusive forces contribute to negative feedback remains to be tested.

How do cells read out these forces to constrain the levels and spatial distribution of leading-edge regulators? In previous work [2], we showed that membrane tension is likely to regulate this long-range inhibitor: increasing membrane tension suffices to inhibit leading-edge signals, whereas decreasing membrane tension prevents their restriction. Similarly, micropipette-based aspiration of plasma membrane at the rear of a migrating keratocyte leads to changes in leading-edge actin dynamics and protrusion velocity within seconds [63]. Such data are consistent with locally applied mechanical forces rapidly propagating through the entire cell. However, it has been difficult to discern whether these mechanical forces depend on membrane tension directly or other correlated physical properties, such as cell shape or local membrane curvature. Recent work has even called into question whether changes in membrane tension can propagate across the cell rapidly enough to serve as a global integrator of mechanical forces [64]. In other instances, it has been possible to discriminate between the effects of multiple mechanical inputs on cell signaling (e.g., substrate stiffness versus cell spread area on yes-associated protein [YAP]/transcriptional co-activator with PDZ-binding motif [TAZ] regulation) through the careful design of conditions capable of decoupling these inputs [65]. Here, we took a similar approach by leveraging a mechanical perturbation (mild hypotonic treatment), which rescued the ability of WAVE-null cells to constrain Rac activity. Importantly, this perturbation does not increase membrane tension (Fig 4C) despite causing significant changes to cell shape—up to a 2-fold increase in volume (Fig 4D). These results disfavor mechanisms in which the mechanosensor restricting Rac activation responds directly to membrane tension, suggesting that tension sensors such as stretch-activated ion channels (e.g., Piezo [64,66]) cannot be the sole class of mechanosensors mediating neutrophil long-range inhibition. Given that osmotically induced swelling and/or shrinking leads to changes in membrane geometry [51,52], mechanosensors responding to changes in membrane shape (e.g., membrane curvature-sensitive Bin/amphiphysin/Rvs (BAR) proteins [67,68]) are likely to be involved. An analogous mechanism operates in budding yeast, in which the highly conserved mammalian target of rapamycin complex 2 (mTORC2) signaling pathway is engaged by proteins sensing changes in plasma membrane geometry [69]. Furthermore, we found in neutrophils that mTORC2 signaling restricts leading-edge polarity in response to cell stretching [18].

Along with generating long-range negative feedback, actin assembly is thought to provide short-range positive feedback to enable polarity [5,70]. However, assessing the role of actin assembly in positive feedback has been challenging. Much of this difficultly has stemmed from a lack of tools for decoupling actin’s role in both types of feedback. Pharmacological inhibitors of actin polymerization like latrunculin target all actin networks and would be expected to break all actin-based feedback (i.e., resulting in pleiotropic effects). Even our more focused approach of targeting only WAVE (Figs 1 and 2) or Arp2/3 complex (Fig 3) initially failed to provide this decoupling: the actin networks assembled by each complex generate mechanical forces at the plasma membrane that are required for long-range negative feedback. It was only when we combined genetic perturbations (i.e., knockout of *HEM1* or *ARP2*) with mechanical perturbations (to generate long-range negative feedback) that we were able to isolate and dissect the role of branched actin assembly in providing short-range positive feedback. This combined approach revealed that branched actin assembly is not required for short-range positive feedback, in contrast to the general assumptions in the field [70–73].

When placed under confinement, cells lacking the WAVE complex revert from having finger-like protrusions to sheet-like protrusions that resemble wild-type morphology (Fig 6A–6D), a result that is captured by even a highly simplified model describing the interplay between actin-based protrusive force and membrane bending energy (Fig C-E in S2 Fig). Our observations that WAVE-null cells polarize and migrate using blebs are consistent with prior work showing that neutrophils and other migratory cells use distinct strategies to achieve migration in different external environments [57,74–77]. However, neutrophils migrating using this bleb-based mode strongly polarize Rac activity to a small section of the plasma membrane (Fig 5B and 5C), and Rac activity coincides with protrusion formation and extension (Fig 6E and 6F). These results suggest that a branched actin-independent feedback circuit organizes the local positive feedback promoting Rac activity during this mode of migration. It is striking that neutrophils may rely on Rac activity to organize motility around both actin-rich and bleb-based protrusions, despite these protrusion types relying on very different mechanisms for their construction and for leading-edge advancement. Other cell types that migrate using blebs, such as zebrafish primordial germ cells (PGCs), rely on Rac activity to support bleb growth [78]. Bleb-based motility in PGCs also relies on cell division cycle 42 (Cdc42)-dependent “wrinkling” of the plasma membrane to create membrane reservoirs that can be released as blebs form and expand [79]. Because the Cdc42/WASP axis supports neutrophil polarity [10,11], it would be interesting to test whether this pathway contributes to membrane wrinkling in neutrophils. Such a mechanism would enable neutrophils to extend large amounts of membrane stores when moving through complex microenvironments in vivo or to buffer themselves against changes in cell volume caused by fluctuations in osmolarity. Rac activity may also perform the same function during both migration modes, for example, by promoting local weakening of MCAs to facilitate leading extension. In neutrophils migrating using actin-rich protrusions, local depletion of MCAs coincides with leading-edge formation [61]. Similarly, bleb formation often relies on disruption of MCAs [80].

In summary, we used a combination of genetic and mechanical perturbations to show that neutrophils use 2 mechanistically distinct programs for generating polarity. The first relies on assembly of branched actin networks at the leading edge and is indispensable for polarization in microenvironments in which adhesion to substrate is required for motility. However, in microenvironments in which neutrophils experience compression forces, a second hidden bleb-based program can maintain polarity even if the branched actin-dependent program is broken. By decoupling changes in membrane tension from changes in cell volume, we further show that polarity-regulating mechanosensors are unlikely to respond directly to tension changes but may instead distinguish changes in membrane morphology, such as local curvature [81]. Going forward, leveraging such well-defined environmental perturbations will be helpful in deconvolving the pleiotropic effects that arise when disrupting components with multiple cellular functions. This strategy will be particularly important for dissecting processes such as directed migration, in which a complex interplay between cell-intrinsic and cell-extrinsic factors underlies cell physiology.

## Methods

### Cell culture

Cell culture was performed essentially as previously described [42]. HL-60s were grown in RPMI 1640 media supplemented with L-glutamine and 25 mM HEPES (Corning; Corning, NY) and containing 10% (v/v) heat-inactivated fetal bovine serum (Gibco; Waltham, MA). Cultures were maintained at a density of 0.2–1.0 million cells/mL at 37°C/5% CO_2_. dHL-60s were obtained by adding 1.5% (v/v) DMSO (Sigma-Aldrich; St. Louis, MO) to actively growing cells at a density of 0.3 million/mL followed by incubation for an additional 4–5 d. HEK293T cells (used to generate lentivirus for transduction of HL-60s) were grown in DMEM (Corning; Corning, NY) containing 10% (v/v) heat-inactivated fetal bovine serum (Gibco; Waltham, MA) and maintained at 37°C/5% CO_2_.

### Plasmids

A vector for mammalian expression of PakPBD-mCitrine was generated by PCR amplification of previously described PakPBD [7] and mCitrine, and the PCR products were ligated into the pHR backbone between the MluI and NotI sites. Guide RNAs (gRNAs) with homology to exon 5 of *NCKAP1L/HEM1* (5′ TGTCACGGATTGAAGATCGG 3′) and exon 1 of *ACTR2* (5′ GGTGTGCGACAACGGCACCG 3′) were cloned into the previously described LentiGuide-Puro vector [82], Addgene plasmid #52963. A vector expressing human-codon–optimized *Streptococcus pyrogenes* Cas9-BFP was previously described [42]. To generate a second knockout of *HEM1* (i.e., for the “WAVE-null, clone 2” cells), a gRNA with homology to exon 2 of *NCKAP1L/HEM1* (5′ GAAAGGTGGCTTAGATTTG 3′) was used.

### Transduction of HL-60s

Transduction of HL-60s was performed essentially as previously described [42]. HEK293T cells were seeded into 6-well plates and grown until approximately 80% confluent. For each well, 1.5 μg pHR vector (containing the appropriate transgene), 0.167 μg vesicular stomatitis virus-G vector, and 1.2 μg cytomegalovirus 8.91 vector were mixed and prepared for transfection using TransIT-293 Transfection Reagent (Mirus Bio; Madison, WI) per the manufacturer’s instructions. Following transfection, cells were grown for an additional 3 d, after which virus-containing supernatants were harvested and concentrated approximately 40-fold using Lenti-X Concentrator (Clontech; Mountainview, CA) per the manufacturer’s instructions. Concentrated viruses were frozen and stored at −80°C until needed. For all transductions, thawed virus was mixed with approximately 0.3 million cells in growth media supplemented with polybrene (8 μg/mL) and incubated overnight. Cells expressing desired transgenes were isolated by culturing in growth media supplemented with puromycin (1 μg/mL) or using fluorescence-activated cell sorting (FACS) as appropriate (FACSAria2 or FACSAria3; BD Biosciences; Franklin Lakes, NJ).

### Generation of knockout cell lines using CRISPR/Cas9

Wild-type HL-60s were transduced with vectors containing puromycin-selectable gRNAs targeting *HEM1/NCKAPL1* or *ARP2/ACTR2*. Following selection, cells were then transduced with an *S*. *pyrogenes* Cas9 sequence fused to tagBFP. Cells expressing high levels of Cas9-tagBFP were collected using FACS, after which a heterogeneous population was obtained, as assessed by immunoblot and by sequencing of the genomic DNA flanking the Cas9 cut site. These cells were then diluted into 96-well plates at a density of approximately 1 cell per well to generate clonal lines, which were again verified by genomic DNA sequencing and immunoblot. We verified that candidate clonal lines arose from single cells as previously described [42].

### Preparation of dHL-60s for microscopy

For experiments in which cells were observed in unconfined environments, 96-well #1.5 glass-bottom plates (Brooks Life Sciences; Chelmsford, MA) were coated with a 100 μL solution of 20 μg/mL porcine fibronectin (prepared from whole blood) and 0.4 mg/mL bovine serum albumin (BSA; endotoxin-free, fatty acid free; A8806, Sigma; St. Louis, MO) dissolved in Dulbecco’s Phosphate Buffered Saline (DPBS; 14190–144, Gibco; Waltham, MA) and incubated for 30 min at room temperature. The fibronectin solution was then aspirated, and each well was washed twice with 300 μL DPBS. For each well, 0.2–0.4 mL of dHL-60s in growth media were pelleted at 200*g* for 5 min, resuspended in 100 μL imaging media (Leibovitz's L-15 with 0.2% [w/v] BSA, 0.4 mM NaOH, and 1 nM fMLP) and plated. For experiments in which PakPBD polarity was quantified (e.g., Fig 2B–2F) or in which trajectories were measured (Fig 1C–1E), 5 μM CellTracker Red (C34552, Thermo Fisher) or 1 drop/500 mL NucBlue (R37605, Thermo Fisher), respectively, were added to the imaging media. Cells were then incubated at 37°C/5% CO_2_ for >10 min to permit adherence to the glass, followed by one wash using 100 μL imaging media (containing neither CellTracker Red nor NucBlue dyes).

For experiment in which cells were observed in confined environments (i.e., using PDMS-based devices), 25-mm round #1.5 glass coverslips were coated/incubated with a fibronectin/BSA solution (as described in the preceding paragraph) and were washed once with 1 mL DPBS and once with 0.4 mL deionized water. Immediately prior to plating cells, coverslips were dried under gaseous N_2_. For each coverslip, 0.2–0.4 mL of dHL-60s in growth media were pelleted at 200*g* for 5 min, resuspended in 20 μL imaging media with 5 μM CellTracker Red, plated, and incubated at 37°C/5% CO_2_ for >10 min to permit adherence to the glass. Cells were then washed once using 50 μL imaging media (without CellTracker Red) and 20 μL imaging media with 10 μM nv-fMLP (chemically caged fMLP, prepared as in [54]). PDMS devices (see next section for fabrication) were then placed on top of cells as depicted in Fig 5A, leftmost panel.

### Microscopy hardware

All imaging experiments with the exception of those depicted in Fig 1B–1E and Fig B in S1 Fig were performed at 30°C on a Nikon Eclipse Ti inverted microscope equipped with a motorized laser total internal reflection fluorescence (TIRF) illumination unit, a Borealis beam conditioning unit (Andor), a CSU-W1 Yokugawa spinning disk (Andor; Belfast, Northern Ireland), a 60X PlanApo TIRF 1.49 numerical aperture (NA) objective (Nikon; Toyko, Japan), an iXon Ultra EMCCD camera (Andor), and a laser merge module (LMM5, Spectral Applied Research; Exton, PA) equipped with 405, 440, 488, and 561-nm laser lines. All hardware was controlled using Micro-Manager (UCSF). For experiments performed under confinement, the PDMS devices were connected with PE 20 tubing (Braintree Scientific; Braintree, MA) to a vacuum regulator (IRV1000-01B, SMC Pneumatics; Tokyo, Japan), which, in turn, was connected to the building-wide vacuum supply.

Imaging experiments depicted in Fig 1B–1E and Fig B in S1 Fig were performed at 37°C on a Nikon Eclipse Ti inverted microscope equipped with a motorized stage (ASI), a Lamba XL Broad Spectrum Light Source (Sutter; Novato, CA), 20x 0.75 NA Plan Apo and 60x 1.4 NA Plan Apo objectives (Nikon), and a Clara interline CCD camera (Andor). All hardware was controlled using Nikon Elements.

### Imaging and analysis

ImageJ (NIH), CellProfiler (Broad Institute; https://cellprofiler.org/), RStudio, Excel (Microsoft; Redmond, WA), custom Python code, and Prism (Graphpad; https://www.graphpad.com/) were used for all image analysis.

For the mean square displacement, trajectory, and instantaneous velocity measurements (e.g., Fig 1C–1E), NucBlue-labeled nuclei were imaged using a 20x objective and DAPI filter cube every 15 s for 10 min by widefield epifluorescence microscopy. The NucBlue channel was used to create a binary mask using Otsu’s method, from which the individual nuclei were segmented. For each nucleus, the center of mass of its binary representation was calculated in each frame. Displacements were calculated by measuring the center of each nucleus at each timepoint and calculating the distance from its starting point (i.e., at t = 0 s). A plot of mean square displacement versus time interval was calculated using the “imsd” function in Trackpy (https://soft-matter.github.io/trackpy/v0.3.2). Mean instantaneous velocities were determined by measuring the distance traveled by the segmented nuclei over each time interval and dividing by the duration of the interval. Mean values were calculated from all cells over all time intervals. Cells were omitted from analysis for the following reasons: (i) entering or leaving the field of view during the 10-min observation window, (ii) achieving a maximum displacement of <5 μm during the 10-min observation window (i.e., to remove dead cells and debris), and (iii) undergoing collisions and/or forming clumps that prevented reliable segmentation of individual nuclei. For the trajectory plots depicted in Fig 1C, the x-y coordinates of the nuclear positions were normalized to (0, 0) at 0 s. All summary statistics described in the main body of the Results section are mean ± SEM.

For the polarity measurements (Figs 2B–2F, 3C, 3D, 4E, 4F, 5B, 5C and 6B–6D and S3 Fig and S4 Fig), 488-nm and 561-nm lasers were used to image PakPBD-mCitrine and CellTracker Red, respectively, every 10 s for 5 min by spinning disk confocal microscopy. Focal planes were chosen to be in the lower half of the cell, just above the ventral surface. Cells were stimulated with chemoattractant (fMLP) immediately following the 60 s timepoint. For experiments performed in 96-well plates (unconfined migration) in isotonic media (e.g., Fig 2B–2F), imaging media containing 0.2% BSA and 200 nM fMLP were added to cells 1:1 to yield a final concentration of 100 nM fMLP. For experiments performed in 96-well plates in hypotonic media (Fig 4E and 4F), cells were plated in imaging media as described in “Preparation of dHL-60s for microscopy.” An equal volume of deionized water with 0.2% BSA and 1 nM fMLP was added to cells, followed by incubation for 10 min at 30°C. Afterwards, an equal volume imaging media containing 200 nM fMLP and 0.2% BSA was added to cells, yielding a final concentration of 100 nM. For experiments performed in PDMS devices (confined migration), a 365-nm UV LED flashlight (Americans’ Preferred) was used to photo-uncage nv-fMLP by holding the end of the flashlight 3–5 mm above the top of the PDMS chamber for approximately 15 s. Prior to each experiment, the power output of the flashlight was measured using a Slim Photodiode Power Sensor (S130C, Thor Labs). Flashlight batteries were replaced when the power output dropped below approximately 70% of the initial reading. Prior to analysis, all images were background subtracted using a “dark” image acquired by blocking all light from reaching the camera and averaging 100 exposures. The CellTracker Red channel was then used to create binary masks of the cell bodies using Otsu’s method, and each cell body was eroded inward by approximately 1 μm. Cell bodies were tracked as described in the preceding paragraph to account for cell movement during the experiment. At each timepoint, the cell bodies were used as seeds to identify the PakPBD-mCitrine signal associated with each cell using a previously described propagation algorithm [83]. The distance *(d)* between the center of mass of the PakPBD-mCitrine signal (weighted by fluorescence intensity) and the center of mass of a congruent PakPBD binary image (representing the cell’s “footprint”) were calculated for each cell at each timepoint. To normalize for differences in cell cross-sectional area, the length *(L)* of the major axis of an ellipse that had the same normalized second central moments as the cellular PakPBD signal was determined. The distance *d* was then divided by 0.5*L*, as depicted in Fig 2C, to obtain a PakPBD polarity score ranging from 0 to approximately 1. However, polarity scores greater than 0.3 were rarely observed due to background signal of PakPBD in the cytosol: this cytosolic background biases the weighted center of fluorescence intensity toward the geometric center of the cell, decreasing the resulting polarity score. In all experiments, polarity was quantified at every 10-s interval (beginning at 50 s prior to stimulation with chemoattractant) with the following exception: in experiments in which UV light was used to uncage nv-fMLP, data from the 10 s and 20 s timepoints were always omitted due to excessive background cause by UV light (omitted data indicated by violet bars in Figs 5C, 6C and 6D and Fig C in S4 Fig). Cells were omitted from analysis for the following reasons: (i) touching the edge of the field of view during the 5-min observation window, (ii) undergoing collisions and/or forming clumps that prevented reliable segmentation of individual cell bodies or PakPBD signal, and (iii) failure to express sufficiently high levels of PakPBD-mCitrine to permit reliable segmentation of cell outlines. All summary statistics described in the text are mean ratio ± SEM.

Calculation of polarity based on angular distribution (Fig 2E) was performed similarly to previous implementations (e.g., [44]) on the same raw images used to calculate polarity based on normalized distance: (i) For each cell at each timepoint, we determined the vector from the geometric center of the cell to the weighted center of fluorescence intensity of the PakPBD signal. (ii) For every pixel within the cell at this timepoint, we measured the fluorescence intensity of the PakPBD signal at that pixel and the vector from the geometric cell center to that pixel. The fluorescence intensity of the PakPBD signal at that pixel was then multiplied by the cosine of the angle between this vector and the vector calculated in “i.” (iii) The values calculated in “ii” for a given cell were summed and divided by the total fluorescence intensity of the PakPBD signal in the cell.

For edge velocity and PakPBD fluorescence measurements (Fig 6E and 6F), images were acquired every 1 s for 5 min. Background-corrected PakPBD images were segmented using a 3-step process consisting of Gaussian smoothing, intensity-based thresholding, and distance-transform-based erosion. The threshold and degree of erosion were chosen manually to account for differences in the intensity and polarization of the biosensor in fluorescence images to align the boundary of the binary image with the apparent edge of the cell. To facilitate temporal analysis of edge properties, these boundaries were then fit using a spline interpolation consisting of 1,000 evenly spaced points. The indices of points over time were aligned to minimize the Euclidean distance between point sets in space between consecutive timepoints. This approach allowed for relatively smooth tracking of points along the cell boundary, making temporal comparisons possible (this approach is conceptually similar to a previously described alignment strategy [24]).

Edge velocity at a particular point *P* at time *t* was tracked by calculating the average of the distance transforms of the binary images at times *t-1* and *t+1* and interpolating the value of the signed distance transform at the coordinates of *P*. Edge fluorescence was tracked by interpolating the value of the background-corrected fluorescence image at the coordinates of *P*. Following alignment of the indices of the points, a temporal comparison of protrusion velocity and background-corrected fluorescence was analyzed by Pearson’s cross-correlation function. Per-cell correlation functions were then averaged over multiple cells to give Fig 6F as previously done [84].

To identify bleb turning events (Fig 6G), previously derived edge velocity maps were smoothed with a Gaussian filter, and protrusions were identified by thresholding the smoothed maps at a threshold value of 0.15 μm/s. Connected components were then labeled (accounting for the periodic nature of the maps in the position axis) and analyzed on a per-protrusion basis. The centers of protrusions were tracked by determining the center of mass of the protrusive region at a given timepoint (again, accounting for periodicity in the position axis). Reversals were called when the displacement of the center of mass changed direction between consecutive timepoints. To reduce false positive and negative reversal calls, our analyses were restricted to continuous protrusions that underwent 3 or more reversals during their lifetime. Next, to analyze the spatiotemporal cooccurrence of identified bleb reversal points and “edges” of the Rac signal, the previously derived fluorescence maps were smoothed and segmented using Otsu’s threshold to binarize the signal. Edge positions were extracted from the binary image using the sobel operator. Finally, these analyses were used together to find the minimum distance between each identified turning event and the edge of the zone of Rac activity at the time of reversal.

### AFM

Custom-made chambers or custom-cut 35-mm glass-bottom dishes were coated for 30 min with fibronectin (prepared as described in the preceding section) and washed once with DPBS. dHL-60s were plated on each dish in growth media and allowed to adhere for at least 10 min at 37°C. After, cells were washed with RPMI supplemented with 2% FBS and 10 nM fMLP (experiments in Fig 3A) or RPMI supplemented with 0.2% BSA and 10 nM fMLP (experiments in Figs 1F and 4C).

Olympus BioLevers (k = 60 pN/nm) were calibrated using the thermal noise method and incubated in 2.5 mg/ml Concanavalin A (C5275, Sigma; St. Louis, MO) for 1 h at room temperature. Before the measurements, cantilevers were rinsed in DPBS. Cells were located by brightfield imaging, and the cantilever was positioned at any location over the cell for tether measurement. Cells were not used longer than 1 h for data acquisition. Tethers were pulled using a CellHesion 200 from JPK mounted on an inverted Nikon Ti microscope. Approach velocity was set to 1 μm/s, contact force to 100–300 pN, contact time to 5–10 s, and retraction speed to 10 μm/s. After a 10 μm tether was pulled, the cantilever position was held constant until it broke. Negative and positive forces relate to the angle the cantilever takes, but the sign is arbitrary. By convention, contacting the cell deflects the cantilever toward positive values. Conversely, when the cantilever is pulled downwards by a membrane tether, the values are negative. For experiments involving hypotonic treatment (Fig 4C), tether force was first measured in cells plated in RPMI with 0.2% BSA and 10 nM fMLP. An equal volume of deionized water with 0.2% BSA and 10 nM fMLP was then added to the cells, and tether force in these same cells was measured over a period of 3–15 min following water addition. Analysis of force-time curves was performed using the JPKSPM Data Processing Software. Note that the differences in mean tether force between wild-type cells in Fig 1F versus Fig 3A and in WAVE-null cells in Fig 1F versus Fig 4C arise from systemic error due to the different hardware setups used for performing each set of experiments.

### Volume measurements

dHL-60s were pelleted at 180*g* for 5 min and resuspended at a density of approximately 20,000 cells/mL in RPMI supplemented with 1 nM fMLP. Volumes were measured using a Beckman Coulter Z2 Coulter Counter with a 100-μ aperture probe. The following settings were used: a calibration factor (Kd) of 59.96, a resolution of 256, a gain of 64, and a current of 0.354 mA. Samples of 0.5 mL were measured for each indicated timepoint. At timepoint 0 min, an equal volume of deionized water with 1nM fMLP was added to the sample, and cell volumes were measured at 1-min intervals for 16 min. For each interval, at least 7,000 cells were measured. A Gaussian function was fitted to each volume distribution to determine the mode volume for the time interval. Analysis was performed using SciDAVis (http://scidavis.sourceforge.net/).

### Details of the simulations

The Monte-Carlo simulations of the membrane shape provided in this work follow the scheme described in [38,85,86]. The membrane is described as a triangulated surface, with each node representing either a membrane or a protein domain. In this work, a network is composed of 3,127 nodes, forming a network of 6,250 triangles. Nodes can move and bonds can flip according to the Monte-Carlo (Metropolis) algorithm, within the fixed topology of a sphere and with distance between nodes limited between minimal and 1.7 times’ larger value while implementing self-avoidance. The system is initially thermalized, which is an initial step to ensure a starting random shape that represents the free thermally fluctuating vesicle with freely diffusing proteins. Such a shape, and protein distribution, is then used as an initial state for the simulation that includes the binding interaction between proteins, spontaneous curvature, and the active protrusive force. The energy of the membrane is expressed as the sum of contributions of membrane bending, direct interactions between membrane proteins, and outward protrusive cytoskeletal forces,

$$W=W_{A}+W_{b}+W_{d}+W_{F}$$ (1)

The energy term due to tension is

$$W_{A}=\frac{k_{A}}{2}\sum_{i=1}^{N_{t}}{\left(\frac{a_{i}}{a_{0}}-1\right)}^{2},$$

where *k*_*A*_ is the elastic constant of the membrane and the sum runs over all of *N*_*t*_ triangles of the network, *a*_*i*_ is area of triangle *i*, and *a*_*0*_ is area of a tensionless triangle. For *a*_*0*_, we choose an equilateral triangle, $a_{0}=\sqrt{3}l_{0}^{2}/4$, with side lengths *l*_0_ = (*l*_*min*_+*l*_*max*_)/2. We define membrane tension as the average stretching energy per membrane area, $\sigma =\langle \frac{W_{A}}{A}\rangle$, where *A* is the area of the membrane for a given microstate and brackets denote the canonical ensemble average. The second term in the energy functional is the standard bending energy of the membrane

$$W_{b}=\frac{\kappa }{2}\sum_{i=1}^{N_{v}}{\left(2H_{i}-C_{0,i}\right)}^{2},$$

where *κ* is the bending modulus of the membrane, *H*_*i*_, and *C*_0,*i*_ the mean and intrinsic curvatures at node *i* (where the intrinsic curvature is zero for a network node that has no protein and it has value *c*_0_ for a node occupied by a protein).

The next term in the energy describes the binding between neighboring proteins, with contact energy *w*,

$$W_{d}=-w\sum_{i<j}^{N_{v}}H(r_{0}-r_{ij})$$

which gives a negative (binding) energy for near-neighbors (*w*>0), using the Heaviside function for separations smaller than *r*_0_~*l*_*max*_.

Finally, we have the energy contribution that describes the active force of magnitude *F*, which acts at every protein node toward the outwards normal

$$W_{F}=-F\sum_{i=1}{\hat{n}}_{i}∙{\vec{x}}_{i}$$

where the sum is over all nodes occupied by proteins, with local outwards unit normal ${\hat{n}}_{i}$ and coordinate ${\vec{x}}_{i}$.

The microstates of the membrane are sampled according to the Metropolis algorithm. The probability of accepting the change of the microstate due to vertex move or bond flip is *min*[1,*Exp*(−Δ*E*/*k*_*B*_*T*)], where Δ*E* is the energy change, *k*_*B*_ is the Boltzmann constant, and *T* is absolute temperature. The energy for a given microstate is specified in Eq 1. For each set of parameters, the system is initially thermalized. Ensemble averaging is done over 200 statistically independent microstates.

Fig 1H and Fig B in S2 Fig show results for a membrane at temperature *T*/*T*_0_ = 0.7 where *T*_*0*_ is room temperature (*≈* 300 K), bending modulus *κ* = 20*kT*_0_ with *ρ* = 11% of active proteins with protrusive force *F* = 1*kT*_0_/*l*_*min*_ and direct interaction constant *w* = 1*kT*_0_. Elastic constants (*k*_*A*_) of *1kT*_*0*_ and *10kT*_*0*_ are depicted in Fig 1H and Fig B in S2 Fig, respectively. Fig E in S2 Fig shows results for simulations performed as in Fig 1H with the further addition of 2 parallel surfaces that confine mobility of the membrane in the z direction. Deviation of the vesicle from quasispherical shapes can be characterized conveniently in terms of the asphericity (*A*). To this end, we calculate the gyration tensor, and its three principle eigenvalues (*λ*_1_≥*λ*_2_≥*λ*_3_), as previously described [38,85]. We point out that a one-dimensional object (where *λ*_2_ = *λ*_3_ = 0) leads to *A* = 1, a two-dimensional axisymmetric disk (where *λ*_1_ = *λ*_2_ and *λ*_3_ = 0) gives *A* = 1/4, and a sphere (where *λ*_1_ = *λ*_2_ = *λ*_3_) gives *A* = 0. The asphericity *A* has frequently been used in the past to characterize the shapes of polymers and membranes.

### F-actin staining

Cells were plated in 96-well glass-bottom plates as described in “Preparation of dHL-60s for microscopy” and stimulated using 100 nM fMLP. At indicated timepoints, an equal volume of “fixation buffer” (280 mM KCl, 2 mM MgCl_2_, 4 mM EGTA, 40 mM HEPES, 0.4% BSA, 640 mM sucrose, 7.4% formaldehyde [w/v] [pH 7.5]) was added to each well, and cells were incubated at room temperature for 15–20 min. The fixation solution was then aspirated from each well, and cells were washed once with “intracellular buffer” (140 mM KCl, 1 mM MgCl_2_, 2 mM EGTA, 20 mM HEPES, 0.2% BSA [pH 7.5]); 100 μL “staining buffer” (intracellular buffer + 130 nM rhodamine-phalloidin [ThermoFisher] and 0.2% Triton X-100) was added to each well. Following incubation at room temperature for 45 min, the staining buffer was aspirated from each well, cells were washed once with “intracellular buffer,” and 100 μL intracellular buffer was added to each well. Cells were stored at 4°C until immediately prior to imaging.

### Fabrication of PDMS devices for cell confinement

PDMS devices were manufactured by mixing Sylgard 184 Silicone Elastomer Base and Sylgard 184 Elastomer Curing Agent (#4019862, Dow Corning; Midland, MI) 10:1 (w/w), followed by degassing under vacuum. The exterior suction cup portion of the device was created by placing 3 aluminum rings on a clean silicon wafer in the following order to create concentric circles: (i) a disc of 14 mm diameter and 0.5 mm height; (ii) a ring of 8 mm inner diameter, 14 mm outer diameter, and 5 mm height; and (iii) a ring of 19 mm inner diameter, 40 mm outer diameter, and 7 mm height. Degassed PDMS mixture was poured into this mold, a glass slide was used to scape excess off the top, and the device was baked at 80°C for 1 h. Following removal of the device from the metal ring assembly, a 0.75 mm biopsy punch was used to create a hole in the top of the device where vacuum tubing could be inserted.

The second part of the device was fabricated using a silicon wafer with a regularly repeating array of micropillars (440 μm diameter, 5 μm height, spaced 1 mm apart; as previously described [76]). Degassed PDMS mixture was poured onto the wafer, circular #1.5 coverslips (10 mm diameter) were pressed over top of these patterns using tweezers, and these assemblies were baked at 80°C for 1 h. A metal spatula and polyethylene cell lifters (#3008, Costar) were used to remove the PDMS-coated coverslips from the silicon wafer, and these coverslips were subsequently attached to the central pillar of the suction cup portion of the device, glass side first. Devices were cleaned by sonication in 70% ethanol for 20 min and placed in a 37°C oven to dry. This cleaning procedure was performed each time a PDMS device was used for an experiment.

### phospho-Pak time courses

Performed essentially as previously described [42]: dHL-60s (4–5 d post differentiation) were serum starved by incubation in starvation media (growth media lacking fetal bovine serum) for 45–60 min at 37°C/5% CO_2_ at a density of 1.5 × 10^6^ cells/mL. For hypotonic treatment experiments (Fig 4B), cells were then diluted 1:1 using deionized water and incubated for an additional 10 min. Cells were next stimulated with 10 nM fMLP, and samples were collected at indicated timepoints by mixing 0.5 mL of cells with 0.5 mL ice-cold stop solution (20% [w/v] trichloroacetic acid [TCA], 40 mM NaF, 20 mM β-glycerophosphate). Samples were incubated at 4°C for 1–12 h, after which proteins were pelleted, washed once with 0.7 mL ice-cold 0.5% (w/v) TCA, and solubilized in 2x Laemmli sample buffer (Bio-rad; Hercules, CA). All time courses were performed at approximately 25°C. Samples prepared for validating CRISPR-mediated gene editing (i.e., Fig A, C, and D in S1 Fig) were similarly prepared using TCA precipitation.

### Immunoblot assays

Immunoblot assays were performed essentially as previously described [42]: Protein samples in 2x Laemmli sample buffer (prepared from 0.5–1.0 million cells) were subjected to SDS-PAGE, followed by transfer onto membranes of nitrocellulose (if using LI-COR secondary antibodies) or PVDF (if using HRP conjugate antibodies). With the exception of blots for Hem1, membranes were blocked for approximately 1 h in a 1:1 solution of TBS (20 mM Tris, 500 mM NaCl [pH 7.4]) and Odyssey Blocking Buffer (LI-COR; Lincoln, NE) followed by overnight incubation at 4°C with primary antibodies diluted 1:1,000 in a solution of 1:1 TBST (TBS + 0.2% w/v Tween 20) and Odyssey Blocking Buffer. For Hem1 blots, membranes were blocked for approximately 0.5 h in a solution of TBS with milk (5% wt/vol) followed by overnight incubation at 4°C with Hem1 antibody diluted 1:3,000 in a solution of TBST + milk (5% wt/vol). For most blots, membranes were then washed 3 times with TBST and incubated for 45 min at room temperature with LI-COR secondary antibodies diluted 1:15,000 in Odyssey Blocking Buffer. For blots of CYFIP2 and Hem1, membranes were washed 3 times with TBST and incubated for 45 min at room temperature with HRP conjugate antibodies diluted 1:10,000 in Odyssey Blocking Buffer. Membranes were then washed 3 times with TBST and 1 time with TBS and were imaged using an Odyssey Fc (LI-COR). For blots using HRP conjugate antibodies, membranes were treated with a SuperSignal West Femto kit (Thermo; Waltham, MA), following manufacturer’s instructions, immediately prior to imaging. Analysis was performed using Image Studio (LI-COR; Lincoln, NE) and Excel. For phospho-Pak immunoblots, the ratio of phospho-Pak to total Pak was calculated. These values were then normalized by scaling each relative to the value of wild-type cells at timepoint 0.5 min for cells stimulated with chemoattractant.

Primary antibodies used were Phospho-PAK1 (Ser199/204)/PAK2 (Ser192/197) (Cell Signaling #2605), PAK2 (3B5) (Cell Signaling #4825), WAVE2 (Cell Signaling #3659; Danvers, MA), Abi1 (Cell Signaling #39444), Arp2 (GeneTex #GTX103311; Irvine, CA), CYFIP2 (GeneTex #GTX110897), Hem1 (previously described [12]), and GAPDH Loading Control Antibody (GA1R) (ThermoFisher). Secondary antibodies used were IRDye 680RD Goat anti-Mouse (Li-cor), IRDye 800CW Goat anti-Rabbit (Li-cor), and Novex goat anti-rabbit HRP conjugate (Life Technologies #65–6120; Waltham, MA).

## Supporting information

S1 Data Excel file with values used to make all plots in all figures. (XLSX)

S1 Fig Characterization of CRISPR-edited cells. (A) Immunoblots of wild-type and WAVE-null cells (i.e., lacking Hem1, the hematopoietic-specific core component of WAVE complex). GAPDH was used as a loading control. (B) Persistence measurements. Each point in the distribution represents the displacement of a cell over a 10-min observation window divided by the total distance it traveled over this time interval. *N* = 273 wild-type and 169 WAVE-null cells pooled from 3 independent experiments. The same raw data were used to generate this plot and the plot shown in 1D. *p* = 0.7 unpaired *t* test. (C) Arp2 antibody immunoblots of wild-type and ARP2-null cells. GAPDH was used as a loading control. (D) dHL-60s in the presence or absence of 100 μM CK-666 for 10 min were then stimulated with 10 nM fMLP, and samples were collected and processed for immunoblot. Rac activity was indirectly quantified as in Fig 2A. Each point represents an average of 5 independent experiments, with data for each experiment normalized to cells without CK-666 at 30 s. Error bars, SEM. *p < 0.05 by unpaired *t* test. Right, representative immunoblot. (E) Immunoblots of wild-type and WAVE-null, clone 2 cells, which were generated using a different gRNA sequence. GAPDH was used as a loading control. (F) Rac activity was quantified for chemoattractant-stimulated cells using antibodies targeting phospho-Pak, a downstream readout of Rac activation. Antibodies targeting total Pak were used as loading controls (see the “Immunoblot assays” section of “Methods” for details). Each point represents an average of 4 independent experiments, with data for each experiment normalized to wild-type cells at 30 s, reported as “1.0.” Error bars, SEM. *p < 0.05 by unpaired *t* test. The underlying data for Fig B, D, and F in S1 Fig can be found in S1 Data.
(TIF)

S2 Fig Computational simulations of membrane tension as a function of curvature of actin nucleators and cell geometry as a function of confinement degree. (A) Schematic depicting computational simulations. (B) Membrane tension σ (see “Methods”) as a function of spontaneous curvature, *c*_*0*_, of actin nucleators. Averaging was performed over 200 statistically independent microstates in equilibrium. Actin nucleators, red. Protein-free bilayer, blue. Error bars, SD. Note that the elastic constant of the membrane (*K*_*A*_) is 10-fold larger than in simulations performed in 1H. (C) The proteins are denoted by red, and their active force *F*_*a*_ by the black arrow. (a) Cylindrical protrusions of a free vesicle, (b) flattened protrusions of a squeezed vesicle. (D) A snapshot from a simulation of a vesicle confined between 2 parallel surfaces, a distance *d* apart. The parameters used in the simulation are: *κ* = 80*kT*_0_, *F* = 1*kT*_0_/*l*_*min*_, *K*_*A*_ = 1*kT*_0_, *c*_0_ = 1/(9*l*_*min*_), *w* = 1*kT*_0_, *ρ* = 11%. (E) Ensemble averaged asphericity as a function of distance *d* between 2 parallel plates, as determined from computational simulations. Asphericity is 0 for a sphere, 0.25 for a very thin disc, and 1 for a very thin rod (gray dashed horizontal lines). Black dots indicate asphericity averaged over an ensemble of 500 statistically uncorrelated microstates, and blue bars denote SD. *l*_*min*_ is the edge length of the triangles in the mesh used to cover the surface of the vesicles. d is expressed in units of *l*_*min*_. Representative vesicle snapshots are shown for *d*/*l*_*min*_ = 2, 3.75, and 7. Actin nucleators denoted by red vertices, and protein-free lipid bilayer denoted by blue.
(TIF)

S3 Fig Control for chemoattractant photo-uncaging experiments. Experiments performed as in 5C, but without photo-uncaging of chemoattractant (violet curve). *N* = 131 wild-type cells pooled from 3 independent experiments. Dashed lines, mean polarity of Rac activity. Shaded regions, ±95% CI of the mean Rac polarity. Grey curve, data from wild-type cells subjected to photo-uncaging at approximately 0–15 s duplicated from 5C to aid in comparison. The underlying data can be found in S1 Data.
(TIF)

S4 Fig Weak confinement of WAVE-null or ARP2-null cells restores polarized Rac activity. (A) Schematic for cell confinement experiments. The height of the chamber was set using a vacuum regulator. (B) dHL-60s expressing the Rac biosensor PakPBD were plated on fibronectin-coated glass in media containing 10 μM caged fMLP and imaged every 10 s by confocal microscopy. Chamber height was set as shown in Fig A in S4 Fig. Values in cyan indicate the degree of Rac activity polarization, as described in 2C, for the topmost cell fully inside each panel. (C) Quantification of Rac polarity as described in 2C for cells prepared as in Fig B in S4 Fig. Violet bar indicates when UV was used to photo-uncage caged fMLP (0–20 s). Shaded regions, ±95% CI of the mean Rac polarity. *N* = 122 wild-type cells pooled from 3 independent experiments; 143 WAVE-null cells pooled from 3 independent experiments; and 122 ARP2-null cells pooled from 2 independent experiments. The underlying data for Fig C in S4 Fig can be found in S1 Data.
(TIF)

S5 Fig Location of bleb reversals suggests that Rac sets permissive zone for bleb propagation. (A) Analysis of Pearson correlation between edge velocity and PakPBD fluorescence as a function of temporal offset in fluorescence. The peak Pearson correlation occurs when fluorescence values of Rac activation are shifted back in time by 9 s relative to membrane extension for wild-type and WAVE-null cells, respectively. Lines and shaded areas, mean ±95% CI. Data for WAVE-null cells are duplicated from 6F to aid in comparison. *N* = 21 wild-type cells pooled from 3 independent experiments. (B) Kymograph depicting edge velocity map with Rac activity zone overlaid. Computationally identified reversals are indicated with white circles. The underlying data for Fig A in S5 Fig can be found in S1 Data.
(TIF)

S1 Text Analytical theory of the protrusions’ width. (PDF)

S1 Video Migration of wild-type dHL-60s on fibronectin-coated glass in uniform chemoattractant (10 nM fMLP) as in Fig 1B (top panel), imaged by DIC microscopy. Time, mm:ss.
(MP4)

S2 Video Migration of WAVE-null dHL-60s on fibronectin-coated glass in uniform chemoattractant (10 nM fMLP) as in Fig 1B (bottom panel) imaged by DIC microscopy. Time, mm:ss.
(MP4)

S3 Video Wild-type, WAVE-null, and ARP2-null dHL-60s expressing the Rac activity biosensor PakPBD-mCitrine were plated on fibronectin-coated glass, stimulated with 100 nM fMLP, and imaged by confocal microscopy every 10 s (as in Figs 2B and 3C). Each frame represents a single focal plane.
(MP4)

S4 Video Wild-type, WAVE-null, and ARP2-null dHL-60s expressing the Rac activity biosensor PakPBD-mCitrine were plated on fibronectin-coated glass, stimulated with 100 nM fMLP (immediately following the 0 s timepoint), and imaged by confocal microscopy every 10 s (as in Figs 2B and 3C). Each frame represents a single focal plane. Video shows representative fields of view for experiments performed as in S3 Video.
(MP4)

S5 Video Wild-type and WAVE-null dHL-60s expressing the Rac activity biosensor PakPBD-mCitrine were plated on fibronectin-coated glass in hypotonic media (0.5× isotonic), stimulated with 100 nM fMLP, and imaged by confocal microscopy every 10 s (as in Fig 4F). Each frame represents a single focal plane.
(MP4)

S6 Video Wild-type and WAVE-null dHL-60s expressing the Rac activity biosensor PakPBD-mCitrine were plated on fibronectin-coated glass in media containing 10 μM caged fMLP and imaged every 10 s by confocal microscopy (as in Fig 5B and Fig B in S4 Fig). Chamber height was set as shown in Fig 5A (strong confinement) or Fig A in S4 Fig (weak confinement). Each frame represents a single focal plane.
(MP4)

S7 Video WAVE-null dHL-60s expressing the Rac activity biosensor PakPBD-mCitrine imaged by confocal microscopy. Cells were prepared as described in Fig 5A. Confinement of cells was repeatedly alternated between a chamber height of 6 μm and 4 μm. Each frame represents a single focal plane.
(MP4)

S8 Video Simulation of compression of a vesicle as described in Fig E in S2 Fig for *d*/*l*_*min*_ = 2.0. (MP4)

S9 Video WAVE-null dHL-60s expressing the F-actin marker Utr261-mCherry (utrophin) were plated on fibronectin-coated glass in media containing 10 μM caged fMLP and imaged by confocal microscopy. Cells were confined as described in Fig 5A. Each frame represents a single focal plane.
(MP4)

S10 Video WAVE-null dHL-60s expressing the Rac activity biosensor PakPBD-mCitrine were plated on fibronectin-coated glass in media containing 10 μM caged fMLP and imaged by confocal microscopy. Cells were confined as described in Fig 5A. Each frame represents a single focal plane.
(MP4)

S11 Video WAVE-null dHL-60s expressing the Rac activity biosensor PakPBD-mCitrine were plated on fibronectin-coated glass in media containing 10 μM caged fMLP and imaged by confocal microscopy. Cells were confined as described in Fig 5A. Colored lines indicate cell boundaries (see “Methods” for details). Images were acquired at 1-s intervals.
(MP4)

S12 Video Wild-type dHL-60s expressing the Rac activity biosensor PakPBD-mCitrine were plated on fibronectin-coated glass in media containing 10 μM caged fMLP and imaged by confocal microscopy. Cells were confined as described in Fig 5A. Each frame represents a single focal plane.
(MP4)

S13 VideoSimulation of compression of a vesicle as described in Fig E in S2 Fig for $\frac{d}{l_{min}}=3.75$.(MP4)

S14 Video WAVE-null dHL-60s expressing the Rac activity biosensor PakPBD-mCitrine imaged by confocal microscopy. Cells were prepared as described in Fig 5A, except the height of the chamber set to 5 μm. Each frame represents a single focal plane.
(MP4)

## Abbreviations

Arp2/3: actin-related protein 2/3
AFM: atomic force microscopy
BAR: Bin/amphiphysin/Rvs
BSA: bovine serum albumin
Cdc42: cell division cycle 42
dHL-60: differentiated HL-60 cell
DPBS: Dulbecco’s Phosphate Buffered Saline
FACS: fluorescence-activated cell sorting
fMLP: N-Formylmethionine-leucyl-phenylalanine
GAP: GTPase-activating protein
gRNA: guide RNA
GTP: guanosine triphosphate
HEM1: hematopoietic protein 1
MCA: membrane-to-cortex attachment
mTORC2: mammalian target of rapamycin complex 2
NA: numerical aperture
NPF: nucleation-promoting factor
Pak: p21-activated kinase
PBD: Pak binding domain
PDMS: polydimethylsiloxane
PGC: primordial germ cell
Rac: Ras-related C3 botulinum toxin substrate
RNAi: RNA interference
ROCK: Rho-associated protein kinase
TAZ: transcriptional co-activator with PDZ-binding motif
TCA: trichloroacetic acid
TIRF: total internal reflection fluorescence
Utr261: utrophin 261
WASP: Wiskott–Aldrich syndrome protein
WAVE: WASP-family verprolin homologous protein
YAP: yes-associated protein
