# Supplementary figures and images for: Cell confinement reveals a branched-actin independent circuit for neutrophil polarity
Source: PLoS Biol. 2019 Oct 10;17(10):e3000457. doi: 10.1371/journal.pbio.3000457 (PMC6805013; doi:10.1371/journal.pbio.3000457)

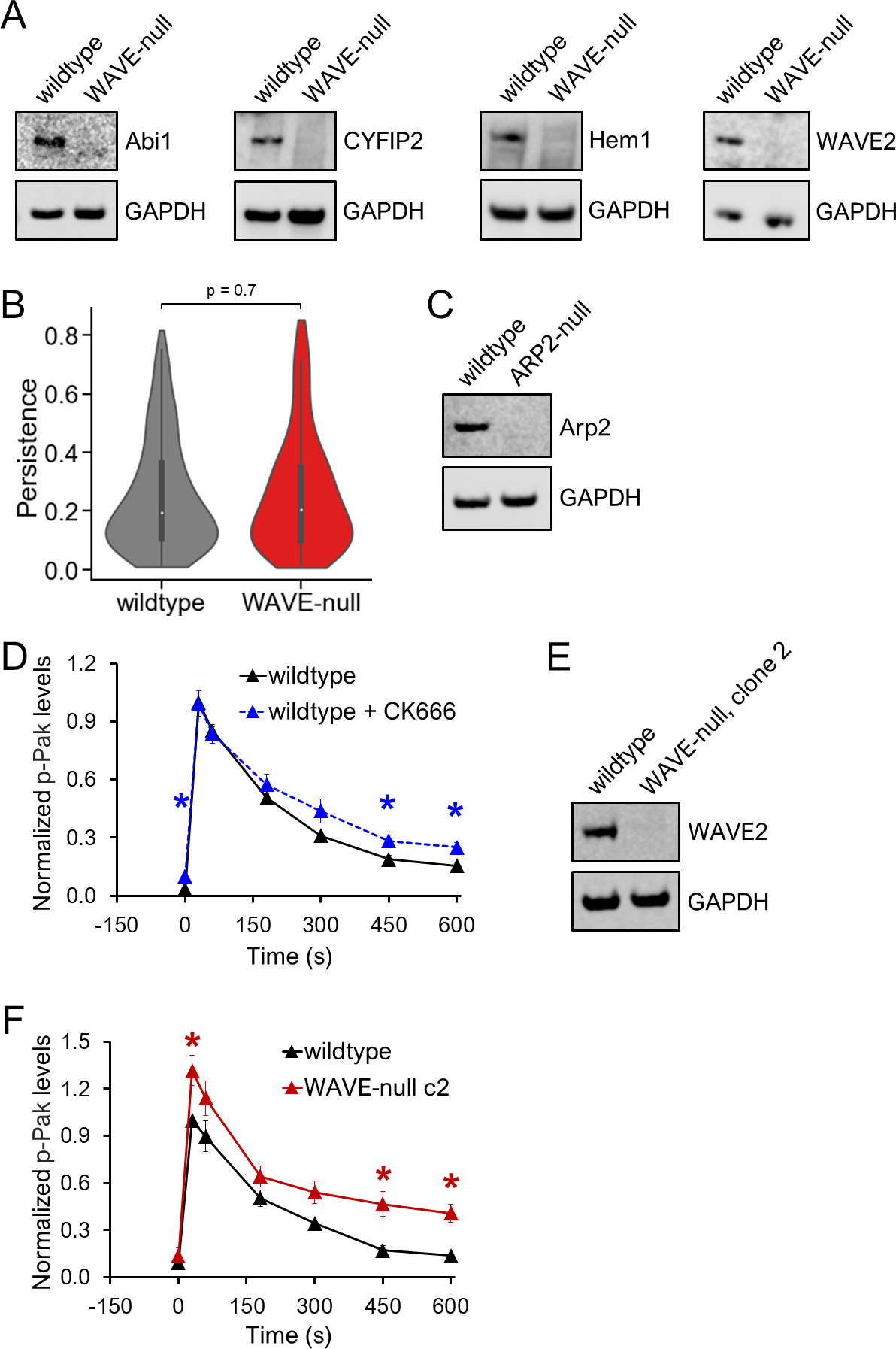

Supplement: S1 Fig — (A) Immunoblots of wild-type and WAVE-null cells (i.e., lacking Hem1, the hematopoietic-specific core component of WAVE complex). GAPDH was used as a loading control. (B) Persistence measurements. Each point in the distribution represents the displacement of a cell over a 10-min observation window divided by the total distance it traveled over this time interval. N = 273 wild-type and 169 WAVE-null cells pooled from 3 independent experiments. The same raw data were used to generate this plot and the plot shown in 1D. p = 0.7 unpaired t test. (C) Arp2 antibody immunoblots of wild-type and ARP2-null cells. GAPDH was used as a loading control. (D) dHL-60s in the presence or absence of 100 μM CK-666 for 10 min were then stimulated with 10 nM fMLP, and samples were collected and processed for immunoblot. Rac activity was indirectly quantified as in Fig 2A. Each point represents an average of 5 independent experiments, with data for each experiment normalized to cells without CK-666 at 30 s. Error bars, SEM. *p < 0.05 by unpaired t test. Right, representative immunoblot. (E) Immunoblots of wild-type and WAVE-null, clone 2 cells, which were generated using a different gRNA sequence. GAPDH was used as a loading control. (F) Rac activity was quantified for chemoattractant-stimulated cells using antibodies targeting phospho-Pak, a downstream readout of Rac activation. Antibodies targeting total Pak were used as loading controls (see the “Immunoblot assays” section of “Methods” for details). Each point represents an average of 4 independent experiments, with data for each experiment normalized to wild-type cells at 30 s, reported as “1.0.” Error bars, SEM. *p < 0.05 by unpaired t test. The underlying data for Fig B, D, and F in S1 Fig can be found in S1 Data. (TIF) [file pbio.3000457.s002.tif]

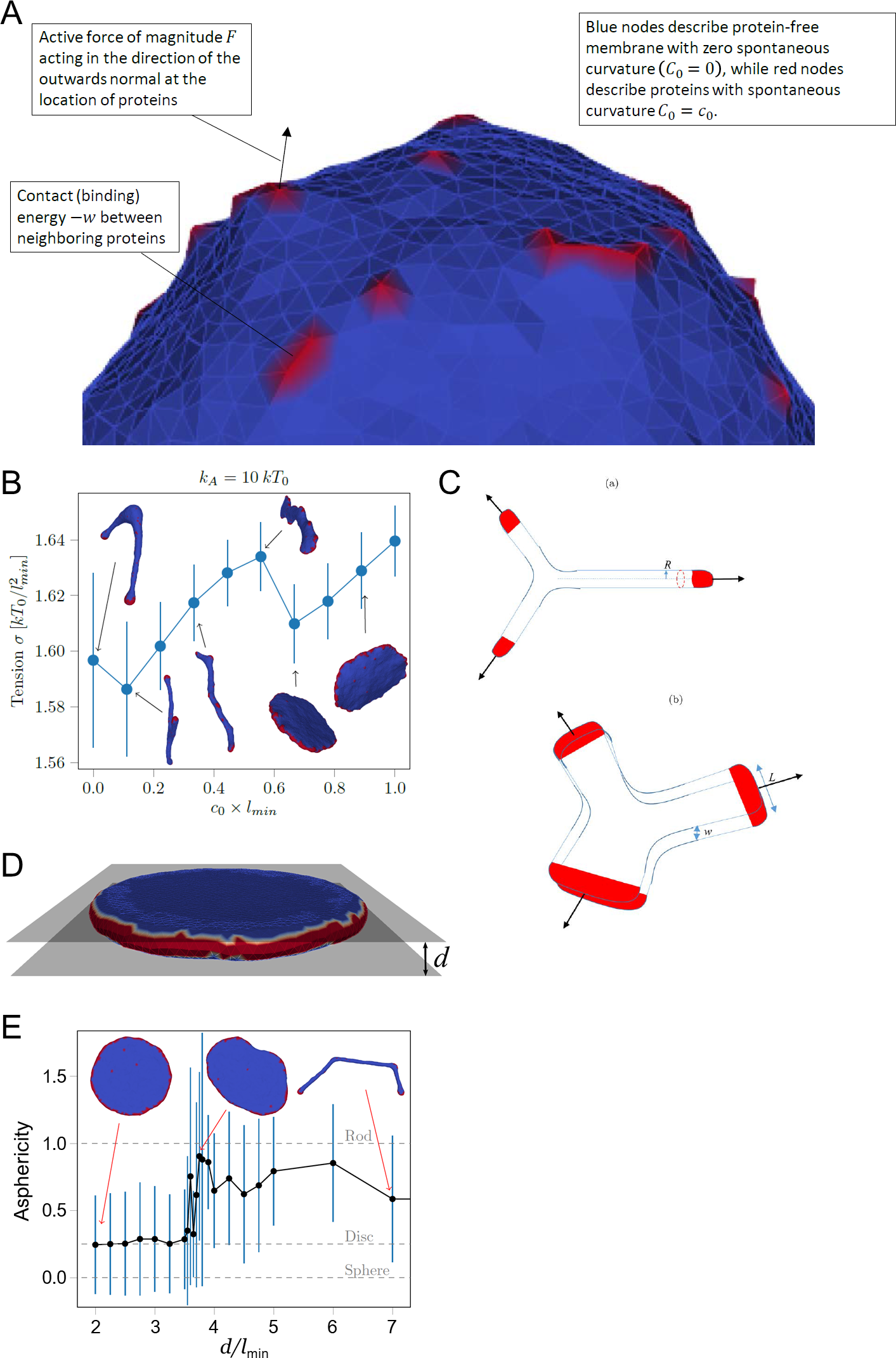

Supplement: S2 Fig — (A) Schematic depicting computational simulations. (B) Membrane tension σ (see “Methods”) as a function of spontaneous curvature, c0, of actin nucleators. Averaging was performed over 200 statistically independent microstates in equilibrium. Actin nucleators, red. Protein-free bilayer, blue. Error bars, SD. Note that the elastic constant of the membrane (KA) is 10-fold larger than in simulations performed in 1H. (C) The proteins are denoted by red, and their active force Fa by the black arrow. (a) Cylindrical protrusions of a free vesicle, (b) flattened protrusions of a squeezed vesicle. (D) A snapshot from a simulation of a vesicle confined between 2 parallel surfaces, a distance d apart. The parameters used in the simulation are: κ = 80kT0, F = 1kT0/lmin, KA = 1kT0, c0 = 1/(9lmin), w = 1kT0, ρ = 11%. (E) Ensemble averaged asphericity as a function of distance d between 2 parallel plates, as determined from computational simulations. Asphericity is 0 for a sphere, 0.25 for a very thin disc, and 1 for a very thin rod (gray dashed horizontal lines). Black dots indicate asphericity averaged over an ensemble of 500 statistically uncorrelated microstates, and blue bars denote SD. lmin is the edge length of the triangles in the mesh used to cover the surface of the vesicles. d is expressed in units of lmin. Representative vesicle snapshots are shown for d/lmin = 2, 3.75, and 7. Actin nucleators denoted by red vertices, and protein-free lipid bilayer denoted by blue. (TIF) [file pbio.3000457.s003.tif]

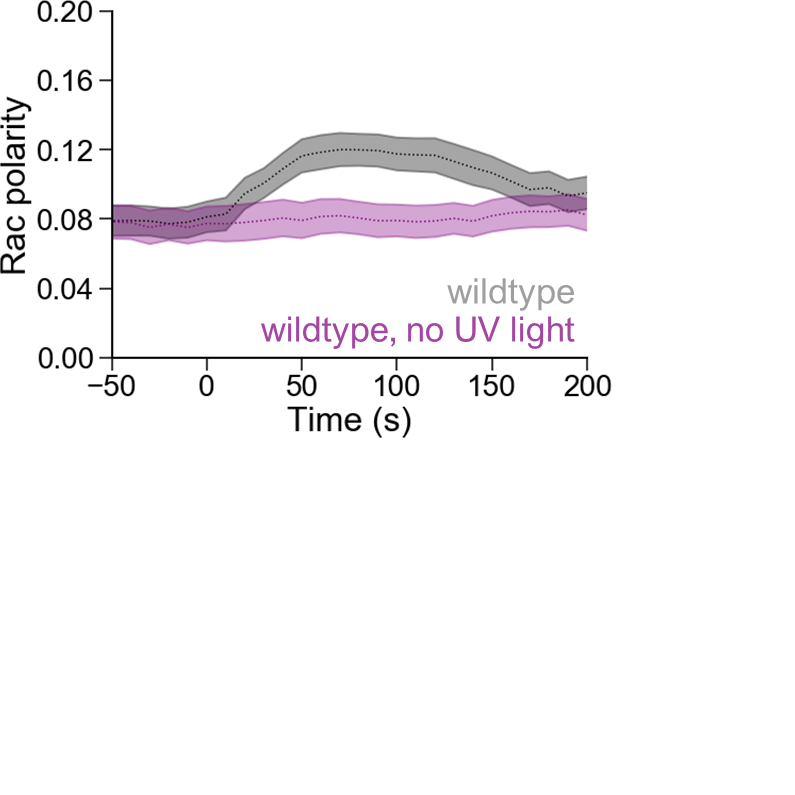

Supplement: S3 Fig — Experiments performed as in 5C, but without photo-uncaging of chemoattractant (violet curve). N = 131 wild-type cells pooled from 3 independent experiments. Dashed lines, mean polarity of Rac activity. Shaded regions, ±95% CI of the mean Rac polarity. Grey curve, data from wild-type cells subjected to photo-uncaging at approximately 0–15 s duplicated from 5C to aid in comparison. The underlying data can be found in S1 Data. (TIF) [file pbio.3000457.s004.tif]

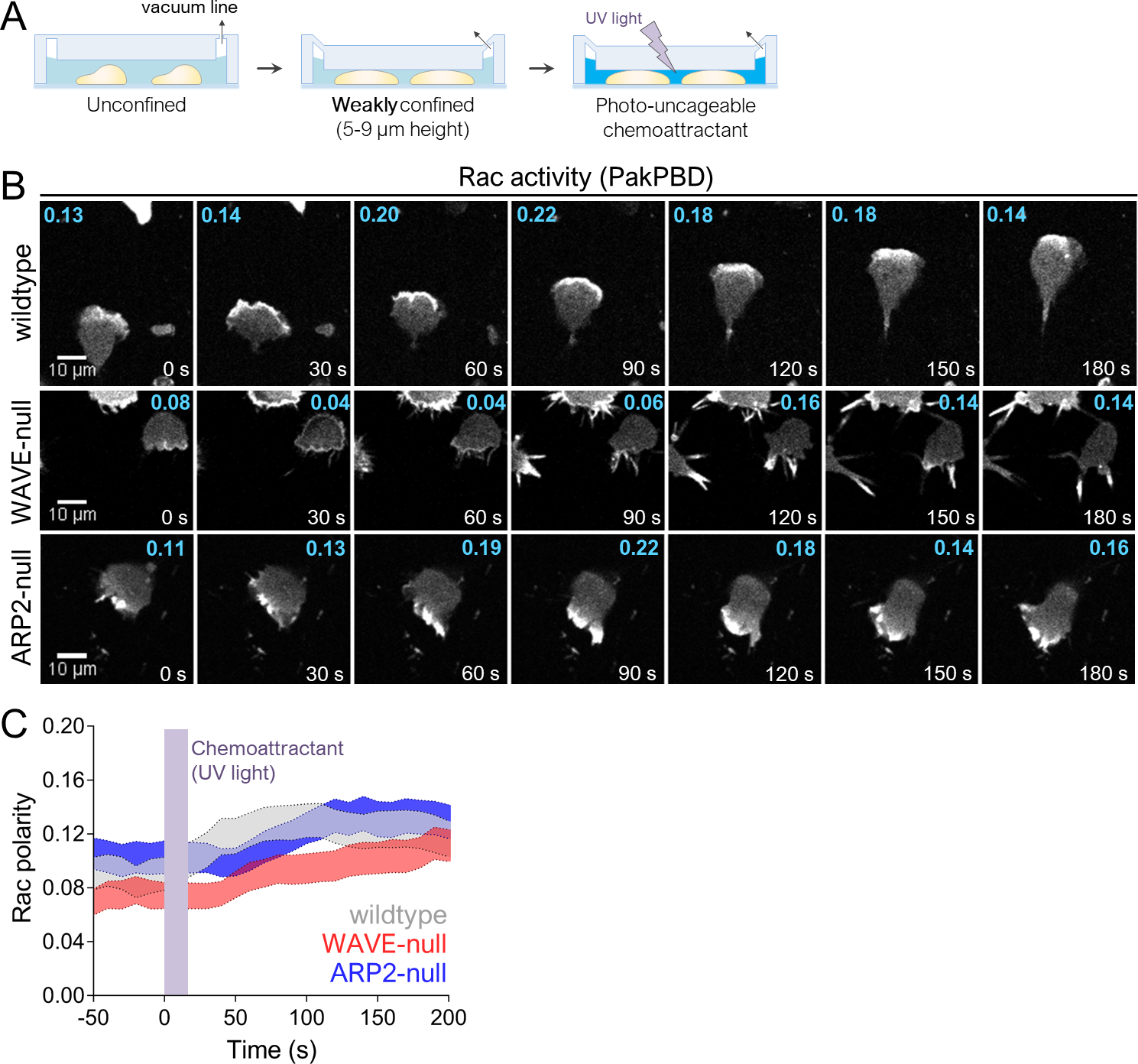

Supplement: S4 Fig — (A) Schematic for cell confinement experiments. The height of the chamber was set using a vacuum regulator. (B) dHL-60s expressing the Rac biosensor PakPBD were plated on fibronectin-coated glass in media containing 10 μM caged fMLP and imaged every 10 s by confocal microscopy. Chamber height was set as shown in Fig A in S4 Fig. Values in cyan indicate the degree of Rac activity polarization, as described in 2C, for the topmost cell fully inside each panel. (C) Quantification of Rac polarity as described in 2C for cells prepared as in Fig B in S4 Fig. Violet bar indicates when UV was used to photo-uncage caged fMLP (0–20 s). Shaded regions, ±95% CI of the mean Rac polarity. N = 122 wild-type cells pooled from 3 independent experiments; 143 WAVE-null cells pooled from 3 independent experiments; and 122 ARP2-null cells pooled from 2 independent experiments. The underlying data for Fig C in S4 Fig can be found in S1 Data. (TIF) [file pbio.3000457.s005.tif]

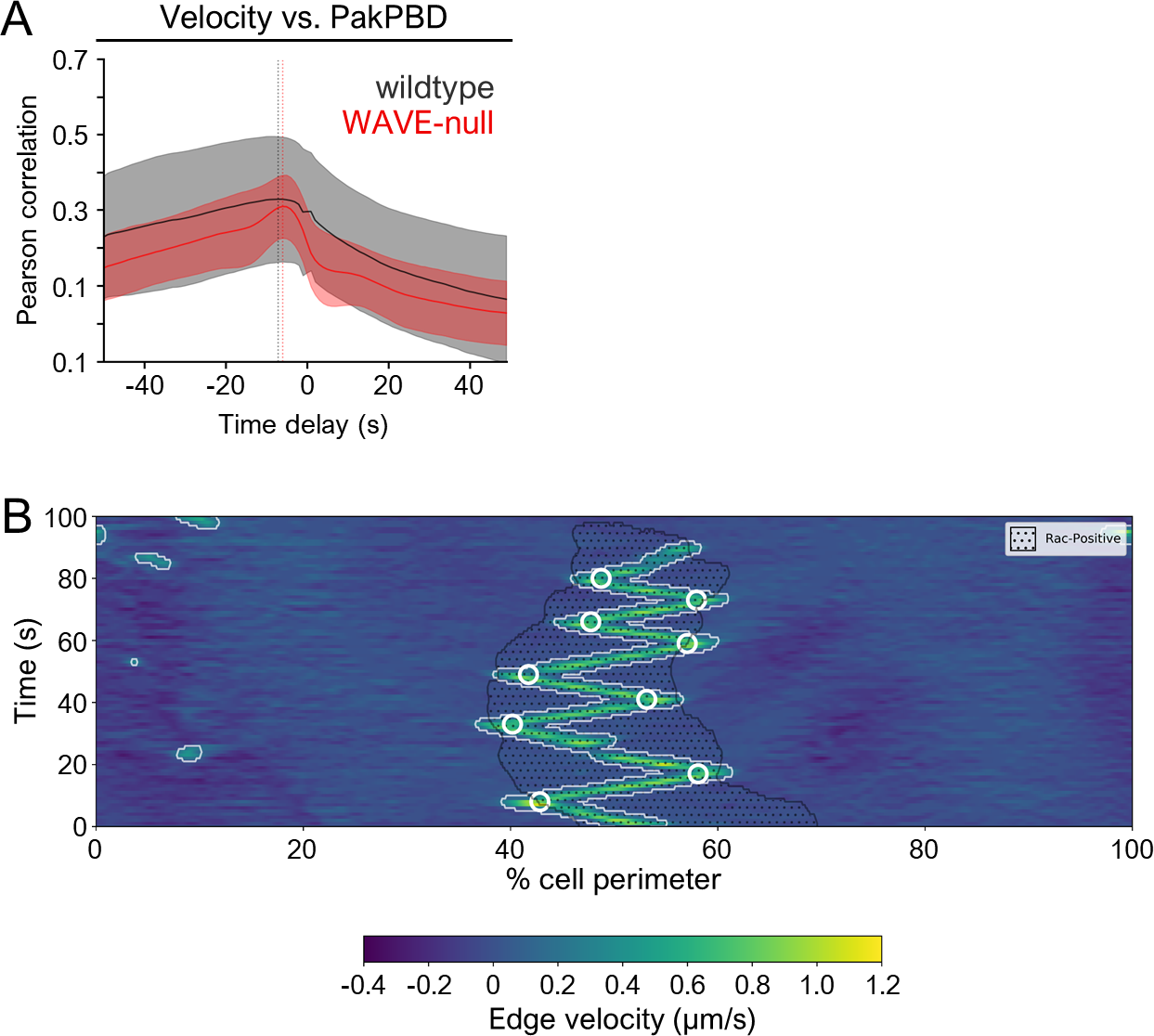

Supplement: S5 Fig — (A) Analysis of Pearson correlation between edge velocity and PakPBD fluorescence as a function of temporal offset in fluorescence. The peak Pearson correlation occurs when fluorescence values of Rac activation are shifted back in time by 9 s relative to membrane extension for wild-type and WAVE-null cells, respectively. Lines and shaded areas, mean ±95% CI. Data for WAVE-null cells are duplicated from 6F to aid in comparison. N = 21 wild-type cells pooled from 3 independent experiments. (B) Kymograph depicting edge velocity map with Rac activity zone overlaid. Computationally identified reversals are indicated with white circles. The underlying data for Fig A in S5 Fig can be found in S1 Data. (TIF) [file pbio.3000457.s006.tif]
