# Supplementary material for: Cell confinement reveals a branched-actin independent circuit for neutrophil polarity
Source: PLoS Biol. 2019 Oct 10;17(10):e3000457. doi: 10.1371/journal.pbio.3000457 (PMC6805013; doi:10.1371/journal.pbio.3000457)
Supplement: S1 Text — (PDF) [file pbio.3000457.s007.pdf]

Cell-extrinsic mechanical forces restore neutrophil polarization in the absence of branched actin assembly: Supplementary Information

Brian R. Graziano[1], Jason P. Town[1], Tamas L. Nagy[1], Miha Fošnarič[4], Samo Penič[3],
Aleš Iglič[3,4], Veronika Kralj-Iglič[5], Nir Gov[6], Alba Diz-Muñoz[2] and Orion D. Weiner[1]
[1] *Cardiovascular Research Institute and Department of Biochemistry and Biophysics, University of California, San Francisco*
[2] *Cell Biology and Biophysics Unit, European Molecular Biology Laboratory, Heidelberg, Germany*
[3] *Department of Theoretical Electrotechnics, Mathematics and Physics, Faculty of Electrical Engineering, University of Ljubljana, Slovenia*
[4] *Laboratory of Clinical Biophysics, Faculty of Medicine, University of Ljubljana, Slovenia*
[5] *Faculty of Health Sciences, University of Ljubljana, Slovenia*
[6] *Department of Chemical and Biological Physics, Weizmann Institute, Rehovot 76100, Israel*

ANALYTIC THEORY OF THE PROTRUSIONS' WIDTH

Before analyzing the shapes of the squeezed vesicles, we can estimate the width of the protrusions of the free vesicle [1].

Width of the protrusions of the free vesicle

When the vesicle is in free solution, the conditions that trigger the transition into the "hydra"-shapes (Fig.S2C(a)) are given by the following force balance:

The force applied at the tip of the cylindrical protrusion by the cluster of active proteins is

$$F_a = f_0 \frac{\pi R^2}{a} \tag{S1}$$

where f_0 is the force per active protein, R is the radius of the cylinder, and a is the area of a protein on the membrane.

This is balanced by the restoring force of the membrane bending energy

$$F_b = \kappa \frac{2\pi}{R} \tag{S2}$$

with κ the bending modulus. The force balance gives the radius of the cylindrical protrusions in this phase of the vesicle shapes

$$R_c = \left(\frac{2\kappa a}{f_0} \right)^{1/3} \tag{S3}$$

This relation, and especially the predicted scaling of the protrusion radius as $(\kappa/f_0)^{1/3}$, was verified by simulations [1]. We next follow a similar calculation to estimate the critical width below which individual protrusions merge to form a continuous cluster along the rim of the confined vesicle.

Protrusions of a squeezed vesicle

When the cylindrical protrusions are now squeezed to a very narrow, and constant height d, it is more natural to define the lateral width of the protrusion L (Fig.S2C(b)). The active force is still given by the number of active proteins at the front of the flat protrusion

$$F_a = f_0 \frac{Ld}{a} \tag{S4}$$

and the bending force

$$F_b = \kappa \frac{2}{d} \tag{S5}$$

The force balance gives the stable lateral width of these flat protrusions

$$L_c = \frac{2\kappa a}{d^2 f_0} \tag{S6}$$

The critical width at which the width of the protrusions merge to form a continuous cluster of proteins along the rim of the squeezed vesicle, can be estimated by substituting in Eq.S6 $L_c \sim R_{vesicle}$. This gives us the following scaling between the critical width and the system parameters

$$d_c \propto \left(\frac{\kappa}{f_0} \right)^{1/2} \tag{S7}$$

Note that due to the two-dimensional geometry, the scaling is different from that of the width of the free protrusions (Eq.S3).

SIMULATIONS OF THE CONFINED VESICLES

Using the system described in Fig.1G (see also Materials and Methods), we observed how a fixed concentration of actin nucleators having zero (or small) spontaneous curvature (which produced vesicles with finger-like protrusions in an unconfined setting) affected vesicle morphology in response to varying degrees of compression. The compression ni the simulations is implemented by defining two parallel surfaces, a distance d apart, that the membranes can not penetrate (Fig.S2D). Surprisingly, we found that increasing levels of confinement (lower values of d/l_{min}) resulted in vesicles converting from a morphology of primarily finger-like protrusions to one containing a single sheet-like protrusion (Fig.S2E, and Videos 8 and 13).

While this simplified model does not account for many other physical properties that neutrophils rely on to regulate shape and polarity (e.g., short range positive feedback, long range inhibition, myosin contractility), it does generally recapitulate our observations in WAVE-null cells where strong compression produced cells with broad smooth protrusions (Fig. 5B, middle row) but more weakly compressed cells retained finger-like protrusions (Fig. S3B, middle row). Furthermore, when WAVE-null cells are subjected to intermediate levels of compression (e.g., ceiling height=5μm), they alternate between forming sheet-like and finger-like protrusions (Video 14), similar to simulations of vesicles at intermediate levels of confinement (Video 13).

Note that the transition in our model, from actin nucleation concentrated within isolated (even if broad) protrusions to a continuous actin rim, could be relevant to the observed formation of blebing activity in the confined WAVE-null cells (Fig.5): for blebing the cells need to have a continuous cortical actin network that can support the formation of significant contractile stresses. Such myosin-driven stresses can then lead to the formation of hydrostatic pressure that results in cytoskeleton-membrane detachment, which is blebing. Therefore, the transition that our model describes in the global organization of the cortical actin, may be relevant to the initiation of blebs, even if contractility is not explicitly described in our model.

Finally, we tested the predicted scaling of Eq.S7 using simulations: Since for $(\kappa/f_0) = 20l_{min}$ we find that the critical width is $d_c \sim 3.5l_{min}$ (Fig.5D), according to the scaling of Eq.S7 we expect the critical width to be:

- $d_c \sim 1.24_{min}$ for $(\kappa/f_0) = 2.5l_{min}$. Indeed in simulations we found that the individual protrusions did not merge even when we squeezed down to $d = 2.25l_{min}$. Below this, the discrete nature of the triangulated surface does not allow for an accurate description of the highly curved rim of the vesicle.

- $d_c \sim 7_{min}$ for $(\kappa/f_0) = 80l_{min}$. Indeed in simulations we found the protrusions to form a continuous cluster at the rim of a uniform circular vesicle for all $d <\sim 7l_{min}$. For $d = 8l_{min}$ we find that the proteins at the rim clearly form several disconnected clusters.

[1] M. Fosnaric, S. Penic, A. Iglič, V. Kralj-Iglic, M. Drab, and N. Gov, Soft Matter (2019).
